# Martini 3 OliGo̅mers: A Scalable Approach for Multimers and Fibrils in GROMACS

**DOI:** 10.1021/acs.jctc.4c00677

**Published:** 2024-08-27

**Authors:** Ksenia Korshunova, Julius Kiuru, Juho Liekkinen, Giray Enkavi, Ilpo Vattulainen, Bart M. H. Bruininks

**Affiliations:** Department of Physics, University of Helsinki, FI-00014 Helsinki, Finland

## Abstract

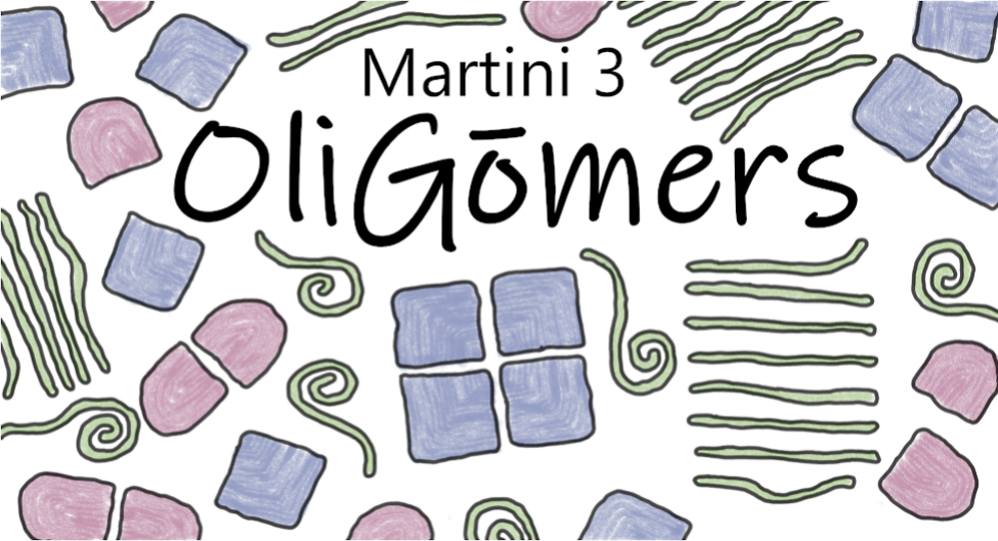

Martini 3 is a widely used coarse-grained simulation method for large-scale biomolecular simulations. It can be combined with a Go̅ model to realistically describe higher-order protein structures while allowing the folding and unfolding events. However, as of today, this method has largely been used only for individual monomers. In this article, we describe how the Go̅ model can be implemented within the framework of Martini 3 for a multimer system, taking into account both intramolecular and intermolecular interactions in an oligomeric protein system. We demonstrate the method by showing how it can be applied to both structural stability maintenance and assembly/disassembly of protein oligomers, using aquaporin tetramer, insulin dimer, and amyloid-β fibril as examples. We find that addition of intermolecular Go̅ potentials stabilizes the quaternary structure of proteins. The strength of the Go̅ potentials can be tuned so that the internal fluctuations of proteins match the behavior of atomistic simulation models, however, the results also show that the use of too strong intermolecular Go̅ potentials weakens the chemical specificity of oligomerization. The Martini-Go̅ model presented here enables the use of Go̅ potentials in oligomeric molecular systems in a computationally efficient and parallelizable manner, especially in the case of homopolymers, where the number of identical protein monomers is high. This paves the way for coarse-grained simulations of large protein complexes, such as viral protein capsids and prion fibrils, in complex biological environments.

## Introduction

Martini is a general-purpose method for coarse grained (CG) molecular dynamics (MD) simulations, with Martini 3 being its most recent version,^1^ and it has a wide user base in the biomolecular simulation community.^2−9^ The main goal of coarse-graining is to simplify detailed simulation models, and thus enable the study of physical phenomena on larger length and time scales. On the one hand, this is achieved by reducing the number of degrees of freedom, allowing larger systems to be simulated.^10^ On the other hand, coarse-graining enables the study of slow and long-lasting dynamic processes due to a smoother potential energy surface. From a practical point of view, Martini in particular has become a popular coarse-grained simulation method for life sciences and soft matter research, based on its building block concept^9^ and its use with the GROMACS and OpenMM software packages.^11−14^

However, due to the nature of coarse-grained simulation models, a certain caution in their use is appropriate. In atomistic MD simulation models, mainly isotropic potentials are used for nonbonded interactions (CHARMM36,^15^ GROMOS,^16^ OPLS,^17^ etc.), relying on the assumption that the detailed structure of the molecules breaks the radial symmetry at short distances (<1.5 nm). The forms of the potentials used in coarse-grained simulation methods, such as Martini, are similar to those used in atomic-level simulation models, but because much of the molecular structure is simplified, the environment of each particle is much more isotropic at short distances than in the atomic-level description. It follows that in Martini, the directionality of many interactions disappears, and thus, for example, the crystal structure of solids is not described correctly.^18^ For this reason, Martini is mainly designed for simulating soft matter systems in the liquid phase. Although proteins are classified as soft matter, their folded core can still have features characteristic of solid matter, such as a stable, long-lived structure. The lack of anisotropic interactions (i.e., in practice hydrogen bonds) is one of Martini’s main shortcomings, causing instability of the folded protein structure. A pragmatic solution to overcome the instability issue is to add another “layer” of interactions, in which case the aim is to strengthen the stability of protein structures.

Currently, the interactions used in Martini can be combined with several bias potentials to improve the accuracy of the description of higher-order protein structures (secondary, tertiary, and quaternary). Commonly used bias potentials include, in particular, the mapping and bonded interaction bias,^19^ the elastic network (EN) model,^20^ and the Go̅ model.^21−24^ All these methods have very much the same goal of guiding the investigated peptide, protein, or its domain toward a certain structure, which must be known and defined in advance.

In terms of the Martini model, the EN and Go̅ models are the most promising due to their quality and usability. They differ from each other in terms of their implementation, although it is more significant that they are suitable for quite different purposes. The EN model uses bonded interactions, where native contacts are kept stable with the help of harmonic potentials when the distance between two particles is short enough (simple cutoff parameter). Therefore, the Martini + EN model is suitable in the cases where the protein does not fold, its fold is not broken, and the protein complexes cannot assemble or disassemble. The Go̅ model, on the other hand, uses the nonbonded interactions based on the contact map^21,25^—a more sophisticated approach than the aforementioned cutoff criterion. Thus, the Go̅ model is more flexible in terms of applications, as it allows the folding and unfolding of protein structures, and also the assembly and dissociation of protein complexes.

The Go̅ model is therefore a promising method that can be of great use in (bio)molecular simulations, especially in the cases where significant changes can be assumed to occur in molecular conformations or aggregation. To our knowledge, the Go̅ model has been implemented for the Martini model for single- and multichain proteins^21,26^ integrated with the commonly used GROMACS MD software.^11−13^ Originally, it was tested by performing pulling and folding simulations for small (less than 350 residues) proteins and peptides, respectively. However, this implementation does not parallelize well, since the Go̅ bonds are expressed as GROMACS pairs in this implementation. This causes domain decomposition errors when the Go̅ bonds span more than two domains, since the pairs are treated as bonded interactions. This problem was solved by defining the Go̅ model via virtual sites, thus avoiding the need to use pairs in GROMACS. This approach was used in the Martini2.2-Go̅ study to investigate the stability of plant light-harvesting complex II)^23^ and in the Martini 3-Go̅ simulation of zinc superoxide dismutase (SOD1).^22^ Additionally, the recently published study of a range of protein–ligand and protein–protein interactions employs virtual sites in combination with Martini 3.^27^ However, these studies defined Go̅ interactions in a uniform fashion, employing the same interaction strength everywhere in the protein complex.

Using previous work^21−23^ as a basis, in this paper we present and study an extension of the Martini-Go̅ model, where the key added value arises from the ability of Go̅ to describe multichain protein complexes. The technique we describe focuses on Martini 3, but it can also be applied to earlier Martini models. To model the dynamic properties of both tertiary and quaternary structures of protein complexes, we group bonds into intramolecular and intermolecular bonds. We demonstrate the implementation of the Go̅ model by studying insulin in the aqueous phase and aquaporin in the cell membrane environment and finding out how the strength of intramolecular and intermolecular Go̅ potentials is reflected in the stability of these protein systems. We determine the Go̅ potentials that give the most accurate agreement in relation to atomic-level simulations, and at the same time we compare the results given by the Go̅ model to the typical protein structures given by the EN. Additionally, we investigate how the chemical environment, here different solvents, affects the sensitivity of the quaternary structure. Finally, using the self-assembly of an amyloid-β fiber as an example, we show how this implementation of the Go̅ model is suitable for studying self-assembly phenomena with a large number of protein monomers.

## Methods

### Building Simulation Systems: Atomistic and Coarse-Grained Descriptions

#### Aquaporin

The structure of the tetrameric aquaporin (PDB ID: 1J4N) was obtained from the protein database (PDB)^28,29^ and its orientation relative to the bilayer was determined from the OPM Web site.^30^ CHARMM-GUI was used to construct the atomistic protein-bilayer system. The initial size of the system was 15 × 15 × 12.5 nm^3^, which, in addition to the bilayer, contained TIP3P water and 0.15 M NaCl as a solvent. The bilayer consisted of 545 POPC molecules and the 4 aquaporin monomers. The total number of atoms was 261,473.

A coarse-grained Martini 3 description of the aquaporin bilayer system was constructed from the atomistic representation with martinize2^31^ and insane,^32^ and had a size of 30 × 30 × 15 nm^3^, containing 76,150 regular-sized water beads^1^ in 0.15 M salt (NaCl). The coarse-graining of the proteins (aquaporin) is discussed in more detail below. The bilayer contained 2900 phospholipids (POPC). In total, the system contained 114,840 beads, with additional 996 virtual beads of the Go̅ model.

#### Insulin

The insulin structures (PDB IDs: 5BTS, 3W7Y) were obtained from the PDB.^29,33,34^ The atomistic insulin dimer was solvated with 20,803 TIP3P waters and 0.15 M NaCl (and, additionally, 4 Na^+^ ions were added to neutralize the system). The initial system size was 7.42 × 7.42 × 7.42 nm^3^. One of the structures (5BTS) was covalently dimerized, but these bonds were removed.

A coarse-grained insulin dimer model was created following the procedure described above (tetrameric aquaporin), and by adding 15,000 regular-sized water beads^1^ and 4 neutralizing Na^+^-ions, resulting in a system size of 12.27 × 12.27 × 12.27 nm^3^. Also in this case, the coarse-graining of insulin is discussed in more detail below.

The CG insulin dimer in water was also used as a starting point to investigate the effects of the solvent (dimer formation and stability, and dissociation). For this, we created systems with varying concentrations (0, 10, 20, 30, 40, 50, 60, 70, 80 mol %) of ethanol (bead type SP1) by replacing some of the water beads with solvent beads. We scanned through a wide range of intermolecular potential ϵ_inter_ values (M, 1, 2, 3, 4, 5, 6, 7, 8, 9, 10, 15, 20 kJ mol^–1^), which results in a total of 117 systems (9 concentrations ×13 ϵ_inter_ values). Similarly, we investigated other solvents (Martini bead types SN2, SN3, SN4, SN5, SN6, SP2, SP3, SP4, SP5, SP6). In these simulations, we scanned through a smaller range of ϵ_inter_ values (5, 6, 7, 8, 9, 10 kJ mol^–1^) where insulin dimer dissociation was observed to occur, and used only a single solvent concentration of 50 mol %. This resulted in a total of 70 simulation systems (10 bead types times 7 ϵ_inter_ values).

#### Amyloid-β

The pentameric amyloid-β structure (PDB ID: 2BEG) was obtained from the PDB database.^29,35^ The B chain was aligned on the C chain, after which all other chains were removed (leaving a dimer of two B chains). No atomistic simulations were carried out for this system. Martinize2^31^ was used to generate Martini protein topology and structure files based on the atomistic structure file of the B-chain dimer, which were used to generate a Go̅ model for a large number of dimers, reflecting the contacts back on a single chain (the “Snake Oil” implementation described in Supporting Information). There were 3 and 62 intramolecular and intermolecular Go̅ bonds, respectively. The models were solvated in water (bead type W, regular-sized)^1^ and neutralized in physiological salt concentration (0.15 M NaCl) using insane.^32^

The translational and rotational matrix of the alignment was used to recursively expand the dimer to a 27-mer fiber made of B chains. The initial fiber system size was 15.77 × 15.77 × 15.77 nm^3^.

For the self-assembly, 8 monomers were placed in a regular grid (2 × 2 × 2). The initial self-assembly system size was 11.15 × 11.15 × 11.15 nm^3^.

### Coarse-Graining of Proteins

#### Martinization

The atomistic 1J4N (aquaporin) and 5BTS/3W7Y (insulin dimer) structures were coarse-grained using martinize2^31^ with Martini3.0.0.^1^ DSSP^36^ was used to determine the secondary structure, taking the whole oligomer into account.^37^ In all amyloid-β cases, the secondary structure was set to a beta-coil-beta motif (CEEEEEEEEESCCSEEEEEEEEEEEEC) using the *-ss* flag in martinize2.^31^ The *-scfix* and *-cys auto* settings were used as recommended by the latest martinize2 protein tutorial available at the cgmartini.nl Web site (http://cgmartini.nl/index.php/2021-martini-online-workshop/tutorials/564-2-proteins-basic-and-martinize-2).

Default martinization: $martinize2 -f aa.pdb -o topol.top -x cg.pdb -dssp/path/to/dssp -p backbone -ff martini3001 -scfix -cys auto.

#### Martinization + EN

The CG models of aquaporin utilized an EN with a force constant of 500 kJ mol^–1^ nm^–2^ with a lower cutoff 0, upper cutoff 0.9 nm, in combination with a bond decay factor 0 and a bond decay power 1.

For the CG models of insulin, which utilized the EN, a force constant of 500 kJ mol^–1^ nm^–2^ was used in combination with the default parameters presented below. In this case, the EN was generated between the two chains, which are covalently linked by the disulfide bridges.

For amyloid-β, the EN was created with an upper cutoff of 1.2 nm to stabilize the horseshoe conformation by creating EN-bonds between the two beta-strands in the beta-coil-beta motif. The EN model was built only for the monomer.

Default martinization + EN: $martinize2 -f aa.pdb -o topol.top -x cg.pdb -dssp/path/to/dssp -p backbone -ff martini3001 -elastic -ef 700.0 -el 0.5 -eu 0.9 -ea 0 -ep 0 -scfix -cys auto.

#### Martinization + Go̅

Two Go̅ setups were constructed for all tested CG protein models. The first one stabilized only intramolecular (tertiary) contacts with Go̅-like interactions (intramolecular Go̅). The second stabilized both intramolecular and intermolecular (tertiary and quaternary) contacts (intra/intermolecular Go̅).

For the intramolecular Go̅ model, the *create_goVirt.py* script, from the 2021 Martini online workshop, was used for each chain separately. As input, the CG structure from martinize2^31^ and an atomistic contact map from http://info.ifpan.edu.pl/~rcsu/rcsu/index.html were used in combination with a lower cutoff of 0.3 nm and an upper cutoff of 1.1 nm.

The intra/intermolecular Go̅ model for the aquaporin and insulin made use of our modified *create_goVirt.py* script, which processes the entire oligomer at once. The new modified script splits the intra- and intermolecular contacts and assigns them separate interaction strengths, ϵ_intra_ and ϵ_inter_. The contact map and coarse-graining were performed on the entire oligomer, using the same settings as the intramolecular Go̅ model, unless specifically stated otherwise.

The intra/intermolecular Go̅ model for the amyloid-β system was produced using the in-house *sorted_goVirt.py* script (“Snake oil”), which analyzes the oligomer assembly, splits contacts to intra- and intermolecular subsets with respective interaction strength values, and finally produces a single chain output containing all parameters required for oligomer assembly (refer to the Supporting Information for implementation details).

#### Control Systems

The parametrization of the CG-Go̅ models was developed in such a way that the goal was the compatibility of the results with the results of the corresponding atomistic models. For comparison, we also investigated CG models based on ENs, using parametrizations that are recommended for the use of ENs in Martini. Based on this, we chose two cases as control systems. In control system 1 (C1), both intramolecular and intermolecular ENs were used. Control system 2 (C2) only has an intramolecular EN. In addition to these, we study control system 3 (C3), based on the previously reported Martini-Go̅ model,^21^ which only has an intramolecular potential of ϵ_intra_ = 12 kJ mol^–1^.

### Simulation Protocols

#### Atomistic Simulations

All atomistic simulations were performed with the GROMACS 2021.X software using the CHARMM36m force field with a 2 fs time step at 312 K for the aquaporin and 310 K for the insulin and 1 atm with periodic boundary conditions. In the aquaporin simulations, we used semi-isotropic pressure coupling, while in the insulin simulations the coupling was isotropic. The Berendsen thermostat was used in equilibration and the Nosé–Hoover thermostat in production runs, both with a time constant of 1.0 ps. During equilibration we used the Berendsen barostat, which was replaced with the Parrinello–Rahman barostat in production simulations, both using a time constant of 5.0 ps and an isothermal compressibility of 4.5 × 10^–5^ bar^–1^. Protein, lipids and solvent (water and ions) were divided into separate temperature groups. The motion of the center of mass was removed every 100 steps. In the Verlet method, the neighbor list was updated every 20 steps with a buffer tolerance of 0.005 kJ mol^–1^ ps^–1^. For van der Waals interactions, we used the force-switch, where rvdw_switch was 1.0, van der Waals cutoff 1.2 nm, and vdw_type was cutoff. Electrostatic properties were treated using Particle mesh Ewald with a 1.2 nm cutoff distance for the real-space part. The leapfrog integrator with the LINCS algorithm to constrain bonds and angles were used. The production runs of aquaporin spanned 1 μs with 3 independent repeats. In production simulations of insulin, the time scale covered 500 ns with 10 repeats.

#### Coarse-Grained Simulations

For all coarse-grained simulations, we used the GROMACS 2021.X software package with the Martini 3.0.0 force field^1^ using a time step of 20 fs at body temperature (312 K for the aquaporin and 310 K for the insulin) and an atmospheric pressure of 1 atm, utilizing periodic boundary conditions. For equilibration, we used the Berendsen barostat with a time constant of 4.0 ps and an isothermal compressibility of 4.5 × 10^–5^ bar^–1^. The pressure coupling was semi-isotropic for the aquaporin system, and isotropic for insulin and amyloid-β. The velocity-rescaling method with a time constant of 1.0 ps served as a thermostat, with a separate coupling for the solute (protein and bilayer, when present) and solvent (water and ions). The Parrinello–Rahman barostat with a time constant of 12 ps and an isothermal compressibility of 3 × 10^–4^ bar^–1^ was used in the production runs. During equilibration, the proteins were position restrained. The motion of the center of mass was removed every 100 steps. The neighbor list was updated with the Verlet method every 20 steps using a buffer tolerance of 0.005 kJ mol^–1^ ps^–1^. van der Waals interactions were modified with a potential-shift Verlet with a van der Waals boundary distance of 1.1 nm. Electrostatic interactions were treated by the reaction field method using a relative permittivity of 15 inside the threshold distance of 1.1 nm and an infinite value elsewhere. The leapfrog algorithm was used for the integration and the LINCS algorithm handled the bond and angle constraints.

In aquaporin simulations, we used 3 independent repeats over 5.5 μs each. Insulin dimer systems were simulated for 5 μs, with 3 repeats. In additional studies of insulin to investigate the effects of solvent, such as ethanol, each system was simulated for 5 μs through 8 repeats with randomized initial velocities, started from a preformed dimer.

### Analysis

For the comparative analysis of atomistic and coarse-grained simulations, the atomistic trajectories were transformed into CG trajectories using martinize2.^31^

#### Native Contacts

The fraction of native contacts refers to the proportion of backbone atom interactions retained from the original crystal (native) structure. It was determined employing the MDAnalysis tool,^38^ based only on protein backbone atoms (BB particles) within a cutoff of 0.6 nm and the coarse-grained protein crystal structure as a reference. To compute the ratio of native contacts to all contacts (native contacts/all contacts), all contacts meeting the same 0.6 nm cutoff between BB particles were counted throughout the simulation. For the aquaporin, the fraction of native contacts was calculated between each neighboring monomers and averaged.

For the amyloid-β contact analysis, native contacts were defined relatively between strands (i.e., excluding intrachain contacts). That is, atom indices are defined starting from zero for each chain, therefore contacts are not chain-specific. The local contact environment (0.6 nm) was compared per atom to the reference structure. The script is added to the Supporting Information paragraph for the Amyloid-β fiber.

#### Binding Curves (Insulin)

The minimum distances between insulin monomers were calculated as time traces during the simulations. The minimum distance traces were discretized into a binary representation specified by a value of 1, if the distance was less than 0.6 nm, and 0 otherwise. The proportion of dimeric proteins was determined by averaging these binary trajectories over all simulation repeats for each system. The results were fitted with a scaled logistic function
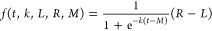
1In this equation, *k* (representing the dissociation rate) and *M* (the half-life) serve as optimization parameters. The parameter *R* sets an asymptotic value to *f* in the limit where *t* tends to infinity, and *L* is the value at time *t* = 0. To estimate errors associated with both binding curves and model parameters, we used Bayesian bootstrapping: we performed 100 bootstrap resampling iterations to obtain bootstrapped fractions of dimeric proteins. The logistic curve was fitted to each resampled fraction.

#### rmsd and RMSF

The root-mean-square deviation (rmsd) of protein structures were calculated with the GROMACS tool gmx rms. The root-mean-square fluctuation (RMSF) of atomic positions in a trajectory were calculated with the GROMACS tool gmx rmsf and VMD.^39^ Experimental (crystal) structures were used as reference based on which the rmsd and RMSF were calculated. The results are shown as averages over all the repeats in each case.

We used RMSF to describe how the parametrization of the EN and Go̅ potentials coupled to the CG models affects protein fluctuations. First, the RMSF was calculated for the atomic-level simulation data of the chosen protein, with the experimentally determined structure of that protein as a reference. From this atomic-level RMSF data, we subtracted the corresponding RMSF calculated from the simulation data of the CG model of the same protein, using the coarse-grained description of the experimental structure of the protein in question as a reference. Due to the differences in the configurational sampling dynamics between the atomic-level and Martini 3 descriptions, finding an exact equivalent in sampling times for the RMSF calculation is a challenging goal. We therefore opted for averaging over the last 20–25% of the production run trajectory in both cases. The choice of the trajectory interval for the CG model is discussed in more detail in the Supporting Information (Figure S3).

## Results

### Aquaporin

**Figure 1:**
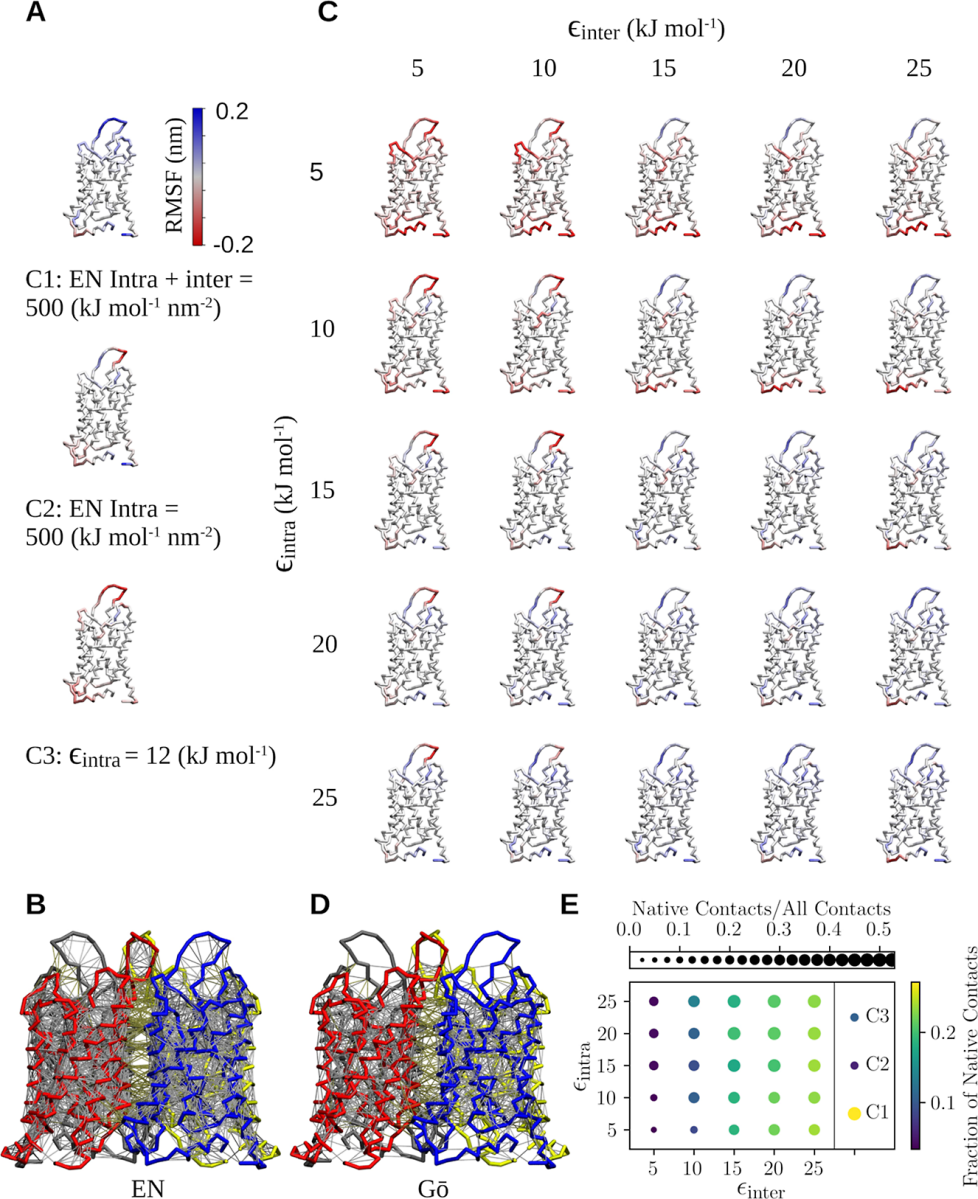
Dependence of aquaporin stability on the parametrization of the Go̅ model. (A) RMSF profiles for control systems of the aquaporin complex: intramolecular and intermolecular EN (C1), intramolecular EN only (C2), intramolecular Go̅ model only (C3). (B) EN visualized for the Coarse-Grained crystal structure of aquaporin tetramer: the four monomers shown in red, blue, yellow, and dark gray, and EN bonds in light gray. (C) RMSF profiles for CG-Go̅ models of aquaporin as a function of Go̅ model parametrization. Color coding indicates the deviation of the RMSF values, with red (blue) corresponding to the CG-Go̅ structure being more flexible (rigid) than the atomistic reference. (D) The Go̅ network visualized for aquaporin tetramer: the four monomers shown in red, blue, yellow, and dark gray, and Go̅ bonds in light gray. (E) The fraction of native contacts as a function of ϵ_intra_ and ϵ_inter_ (in units of kJ mol^–1^), averaged over the last 1 μs of all simulation repeats.

Figure 1A shows structures of aquaporin monomers detached from the tetramer complex using coloring that depicts the difference in the RMSF profile between the atomistic model simulations and the data provided by the CG model. Thus, the blue (red) color describes areas where the fluctuations of the CG model are too weak (strong) compared to the results given by the atomic-level simulations. It can be observed that when the EN is implemented in the CG model of aquaporin (C1, C2), with commonly used interaction parameters, the RMSF data of the protein depends on the parameters used in the EN. Nonetheless, the deviations from the RMSF data based on atomic-level simulations are quite small. The bonds (thin gray lines) in the EN are demonstrated in Figure 1B, which depicts the EN adapted to an entire aquaporin tetramer.

Figure 1C shows similar RMSF profiles for the CG-Go̅ model. For clarity, the results are shown for a monomer detached from the tetramer complex. It is immediately clear that in the CG-Go̅ systems, their RMSF values can be adjusted by varying their interaction parameters ϵ_intra_ and ϵ_inter_. An interesting starting point is the implementation of the Go̅ model typically used until now, where only the intramolecular potential (ϵ_intra_) is included. In this case, fluctuations are overemphasized in part of the protein sequence (C3 in Figure 1A). However, the picture changes when an intermolecular Go̅ potential is included. In this case, with interaction parameters ϵ_intra_ ≥ 15 kJ mol^–1^ and ϵ_inter_ ≥ 15 kJ mol^–1^, the results of the CG-Go̅ models are consistent with the results of the atomic-level simulations.

From the results in Figure 1C, it can be concluded that in the case of the CG-Go̅ model of the aquaporin complex, the stiffness of the various regions is regulated almost exclusively by either ϵ_intra_ or ϵ_inter_. The parameter ϵ_intra_ especially affects the fluctuations within the terminal ends of the monomers, while the parameter ϵ_inter_ affects the stiffness of the loops of the monomers. As in the case of the EN, Figure 1D demonstrates the bonds (thin gray lines) of the Go̅ model adapted to the tetramer complex.

The results of the intermolecular contact analysis in Figure 1E show that the number of native contacts depends on the parametrization of the Go̅ model. The colors of the beads indicate the fraction of native contacts, and their sizes indicate the ratio of native contacts to all contacts. The most obvious effect is given by ϵ_inter_, while the value of ϵ_intra_ has a much smaller effect.

The results are discussed in more detail in a wider context at the end of the paper, together with the results of other investigated proteins.

### Insulin Dimer

The insulin dimer represents an example of a small water-soluble protein assembly with a known dimerization interface and interaction energies.^40^ The stability of the insulin dimer depends primarily on noncovalent interactions between the mostly nonpolar residues at the dimerization interface. Additionally, noncovalent interactions within each individual insulin molecule can affect the stability of the conformations of the insulin monomers and thus the stability of the entire dimer. The choice of Go̅ potentials thus requires careful optimization of both intermolecular and intramolecular interactions, so we systematically studied how the parametrization of the Go̅ model (ϵ_intra_, ϵ_inter_) affects the stability of insulin dimers. For this purpose, we analyzed (i) the stability of the insulin structure (RMSF, rmsd), (ii) the number of intrainsulin and interinsulin native contacts, and (iii) the self-assembly of insulin dimers.

#### Insulin Dimer Stability

**Figure 2:**
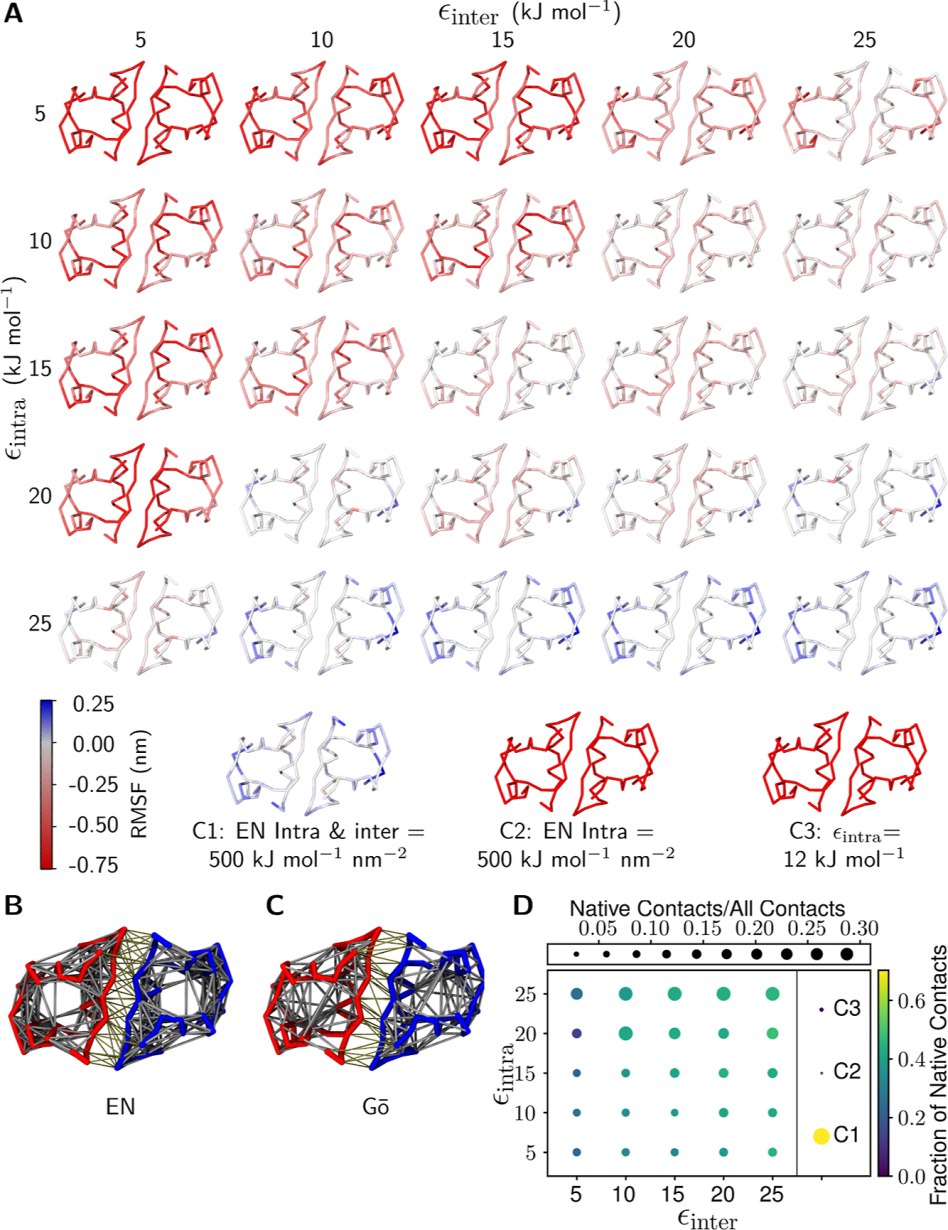
Dependence of insulin dimer stability on Go̅-model parametrization. (A) RMSF values calculated for the insulin (5BTS) dimer, shown as the difference between the atomistic and CG RMSF results. Red coloring indicates that the CG structure is less stable and/or more flexible than the atomistic model, while blue coloring reflects that the CG structure is more rigid than the atomistic description. The dimer was analyzed as a whole molecule. The control systems C1 and C2 include an implementation of the EN, and the control system C3 includes an intramolecular Go̅-potential. (B) The EN and (C) the Go̅ model visualized for the insulin dimer: the two monomers shown in red and blue, and the EN and Go̅ bonds in light gray. (D) The fraction of native contacts shown for a wide range of ϵ_intra_ and ϵ_inter_ values (in units of kJ mol^–1^).

Figure 2A shows that the parametrization of the Go̅ model has a significant effect on the structural fluctuations and the stability of the insulin dimer. Using only an intramolecular potential produces a dimer structure whose fluctuations are too large (see also C3), and the contact at the dimerization interface is not stable. Increasing the strength of the intramolecular potentials alone does not seem to eliminate this problem. In order for the results of the Go̅ model to be in line with the results of the atomic-level model, both ϵ_intra_ and ϵ_inter_ should be about 15 kJ mol^–1^. At lower values, the insulin dimer fluctuates significantly more than the atomistic model, the dimerization interface tends to break, and the insulin molecules dissociate (see Figures 2 and S4). At values above 15 kJ mol^–1^, the CG-Go̅ model of the insulin dimer is too rigid. However, it can be observed from the results that if exact parametrization of the Go̅ model is not possible, it is safer to overestimate rather than underestimate their values.

Based on the control systems (C1, C2), the implementation of the EN is problematic. The resulting dimer structure is either too flexible and unstable, or too stiff. If only the intramolecular potential is considered (C2), the chemical specificity of the polar and nonpolar interactions between the side chains in the Martini-EN model will not hold the insulin molecules together, and the dimer breaks irreversibly in the beginning of the simulations (C2 in Figure S6). Adding the EN between the insulin molecules (C1 in Figure 2A,B) stabilizes the insulin dimer structure so that the dimer does not dissociate, and the RMSF, monomer–monomer distance, and rmsd closely match the behavior of atomistic-level simulations (C1 in Figures S4–S6) and the bonds native to the crystal structure are strictly maintained with the EN (Figure 2D).

The bonds in the Go̅ and ENs (Figure 2B,C) establish contacts whose results shown in Figure 2D bring out that the ratio of contacts increases with increasing ϵ_intra_ and ϵ_inter_.

It should be noted, many proteins have several experimentally determined structures. There are subtle differences in the insulin dimer structures studied here (5BTS, 3W7Y), of which the 3WTY structure leads to a higher dissociation tendency in the CG-Go model simulations when ϵ_inter_ < 10 kJ mol^–1^ (see Figures S5 and S6). However, this tendency disappears as the intermolecular interaction ϵ_inter_ increases, which stabilizes the dimer interface and prevents dimer dissociation.

#### Self-Assembly of Insulin Dimers

How reliably can the Martini-Go̅ model be used when studying molecular self-assembly? We found that even a small ϵ_inter_ allows dimerization of water-soluble proteins. In the case of insulin, we confirmed their dimerization when ϵ_inter_ > 5 kJ mol^–1^ (see Figure S6). However, for the preservation of native contacts, our observations show (Figure 2) that the correct dimerization interface and intramolecular noncovalent bonds are not preserved if ϵ_intra_ and ϵ_inter_ are not carefully chosen.

The results (Figures 2A,D and S4) show that the intramolecular potential ϵ_intra_ should be at least 15 kJ mol^–1^ to maintain the internal stability of insulins, achieving behavior that corresponds to atomistic models. The role of ϵ_inter_ plays the key role, as it helps to stabilize the dimerization interface and the internal structure of the insulin monomers in the dimer.

Comparing the insulin results with those of the aquaporin suggests that the Martini Go̅ simulations of water-soluble molecules require the Go̅ model parameters to have higher values (i.e., more attractive potentials) than in the case of membrane proteins. Especially when studying the dimerization or dissociation of insulin in water, the level ϵ_intra_ > 20 kJ mol^–1^ should be used to maintain the integrity of the intramolecular native contacts and the dimerization interface.

#### Effects of Solvent in Insulin Dimer Stability and Dissociation

Solvents affect protein interactions and structure. Ethanol is a particularly interesting solvent, as its effects on protein structure and functions are well-known and have also been studied for insulin dimer stability.^41^ Ethanol affects protein structure and dimerization primarily by disrupting the noncovalent interactions that maintain the native conformation of proteins. It can also lead to nonspecific aggregation of proteins. Here, we investigate the sensitivity of the Go̅ model to solvent effects (ethanol and others) by varying the level of the intermolecular interaction ϵ_inter_ in our insulin dimer simulations. To this end, we set ϵ_intra_ to 20 kJ mol^–1^ to maintain the structure of individual insulin molecules and scanned through a wide range of ϵ_inter_ values. We focus in particular on (i) the number of native contacts within and between insulins and (ii) the dissociation kinetics and dimerization equilibrium.

**Figure 3:**
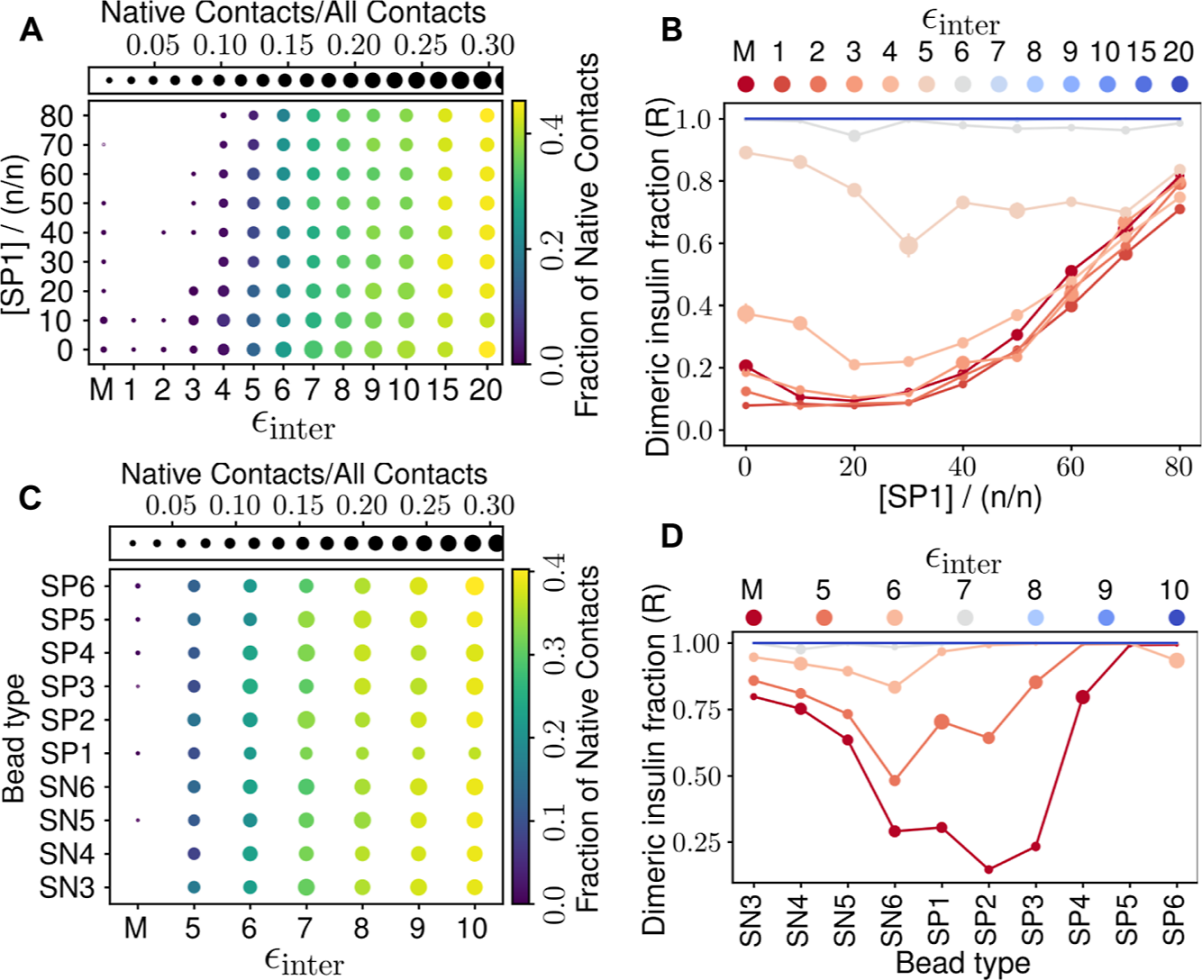
Effects of various solvents on insulin dimer stability. (A) The fraction of native contacts as a function of ethanol (SP1) concentration and ϵ_inter_. The fraction of native contacts is calculated using the coarse-grained insulin dimer crystal structure as the reference with the criterion 0.6 nm between BB beads. Note that ϵ_inter_ = *M* refers to regular Martini monomer–monomer interactions without the Go̅-potential. The colors of the beads indicate the fraction of native contacts, and their sizes indicate the ratio of native contacts to all contacts averaged over the last 1 μs of all repeats. (B) Dimeric insulin fraction (the *R* term in the logistic function) as a function of ethanol concentration, for each tested ϵ_inter_. The dot size indicates the bootstrap error. (C) Same as panel A, but instead of solvent concentration, the *y*-axis is now the bead type. In this data set, the concentration of all solvents is 50 mol %. (D) Same as panel B, but instead of solvent concentration, the *x*-axis is now the bead type. The interaction parameter ϵ_inter_ is given in units of kJ mol^–1^.

As shown in Figure 3A, the fraction of internal native contacts is largely unaffected by ethanol (SP1 bead) concentration but only depends on the ϵ_inter_ value. Figure 3C shows that other tested solvents also have the same behavior. Even at a large intermolecular potential value ϵ_inter_ = 20 kJ mol^–1^, only about half of the native contacts are preserved, and native contacts amount to less than one-half of the total contacts. Fraction of intermonomeric native contacts increase with increasing ϵ, as expected, reaching about 0.5 at ϵ_inter_ = 20 kJ mol^–1^. However, the non-native contacts also increase considerably, as the number of native contacts is no more than 0.3 of the total number of contacts.

To determine the dissociation kinetics (Figure 3B), we computed binding probabilities and fitted the data with a logistic function (see Methods). The values of the parameters *k* and *M* were not statistically different in the fits. However, the *R* parameter, which indicates the dimeric insulin fraction, shows large differences both with different ϵ_inter_ values and ethanol concentrations. Interestingly, increasing the ethanol concentration above the level of 40 mol % increases the dimeric fraction of insulin almost linearly. In addition, when ϵ_inter_ < 4, similar stability is observed at all solvent concentrations as in the Martini model without the Go̅ potential (“*M*” in Figure 3). If the goal is to describe both dimeric and dissociated states, then they are observed only when ϵ_inter_ is set to 4–5 kJ mol^–1^, or to some extent to 6–7 kJ mol^–1^. Above this level, insulin dimers no longer dissociate. When interpreting the results, however, it is good to note that the dimeric insulin fraction is only based on whether there is at least one contact, and it does not indicate whether the dimer is native.

For other solvent types, we performed the same analyses as for ethanol. Figure 3D shows that for them, the native contact information is very similar to that of ethanol, except for the ratio of native contacts to all contacts, which is mostly higher for other solvent types. With the Go̅ model, the results of the dimeric insulin fraction strongly depend on both the solvent type and the intermolecular potential, and it is difficult to observe a general trend. If we focus on the potential ϵ_inter_ = 5 kJ mol^–1^, where ethanol best enables the study of both dimerization and dissociation, then the solvent types SN5, SN6 and SP2 give the best corresponding behavior, while the dissociation tendency is weaker with other solvent types.

### Amyloid-β

One of the main advantages of using the Go̅ model compared to ENs is the ability to study oligomerization and dissociation processes. Amyloid-β fiber is an excellent test case for that purpose. Using the *sorted_goVirt.py* script (“Snake oil”, see the Supporting Information for detailed description), the interaction potentials of the Go̅ model are assigned to a single monomer in a homopolymer system, making it trivial to study such systems with an arbitrary number of monomers. We first determined the most optimal Go̅ model parameter values via the simulations of preassembled fibers, then used those for the self-assembly simulations of the amyloid-β fiber.

#### Preassembled Fiber

**Figure 4:**
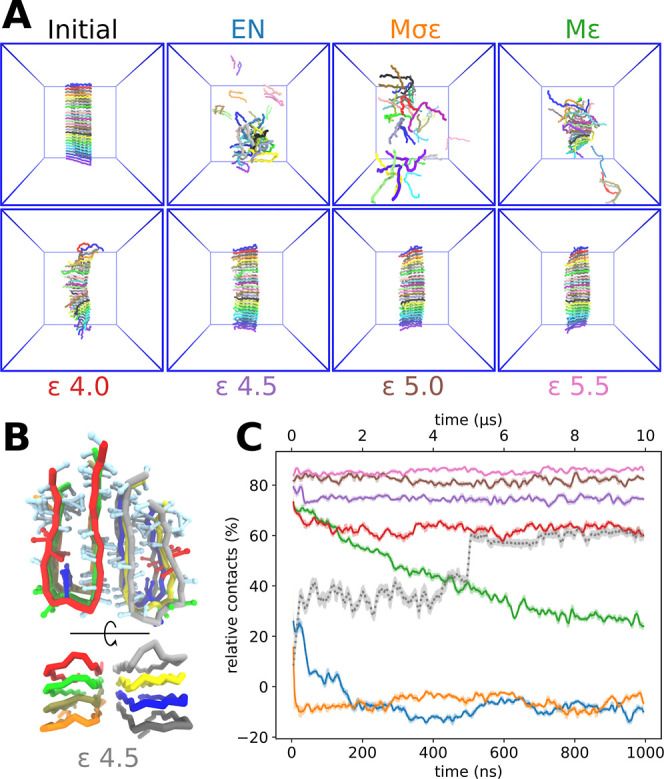
Stability of amyloid-β fiber and its self-assembly (refer to the main text for the naming conventions). (A) The stability of a preassembled amyloid-β fiber after 1 μs with the specified ϵ_inter_ value (see the text). Monomers are color coded. The interaction potentials ϵ are given in units of kJ mol^–1^. (B) Self-assembly of 8 monomers after 10 μs. The top-down view shows the backbone and the side chains colored by residue type: red (negative), blue (positive), green (polar), and light blue (nonpolar). (C) The percentage of relative contacts over time during the simulations. The colors in the graph match the color code of ϵ in panel A. Negative values are possible, because non-native contacts are subtracted from the data. The bottom *x*-axis running up to 1 μs is relevant for the fiber stability assays (not gray). The top *x*-axis with a maximum of 10 μs is for the self-assembly (gray). For an infinite fiber (i.e., every monomer is perfectly in contact with two neighbors), the value of relative contacts reaches 100%. Data shown here have been averaged over a window of 10 ns, and the standard deviation in the sliding average are shown as transparent areas.

Since the minimum number of interactions maintained by the Go̅ model keeps the amyloid model closest to the original Martini, we first determined the minimum level potential required to stabilize the preassembled fiber. To achieve this, we generated 7 models for the amyloid-β monomer (Figure 4A,C). Due to the nature of the “Snake oil” Go̅ approach, the relative order of monomers in the fiber is irrelevant.

The naming convention used for the 7 test models is as follows. The first reference model (“EN” in Figure 4) uses EN intramolecular bonds to maintain the “horseshoe” conformation of the monomer. The other 6 models use the Go̅-type approach for the intramolecular bias with ϵ_intra_ = 12 kJ mol^–1^, where the intermolecular bond distance σ_inter_ or interaction strength ϵ_inter_ is varied. The “*M*σϵ” model uses both σ and ϵ values of Martini 3 in the intermolecular Go̅-bonds. This model thus serves as an intermolecular control: it is otherwise the same as the “EN” model, with the difference that it has intramolecular Go̅ potentials instead of harmonic bonds. The next model (“*M*ϵ”) uses the Go̅ model such that its σ values are obtained from the backbone contact map, but it still uses the default ϵ values of Martini 3 for its intermolecular Go̅ potentials. Finally, we tested four Go̅ models with a parametrization where the intermolecular Go̅ potentials are ϵ_inter_ = 4.0, 4.5, 5.0, or 5.5 kJ mol^–1^.

We start with the control systems. The EN model does not form a stable fiber but rather loosely aggregates and quickly loses the native backbone contacts [Figure 4A (EN model), Figure 4C (blue curve)]. *M*σϵ is comparable to the EN model, but in a way where the monomers are able to sample extended conformations, because the intramolecular Go̅ interactions are relatively weak. The solvated monomers are usually in an extended conformation and only rarely settle into a horseshoe shape [Figure 4A (*M*σϵ model), Figure 4C (orange curve)]. In *M*ϵ, the preassembled fiber does not fall apart as fast as in the EN and Mσ models, but is still unable to maintain a stable fiber [Figure 4A (*M*ϵ model), Figure 4C (green curve)]. In summary, none of these control systems can maintain a stable fiber.

The remaining models use σ and ϵ obtained from the contact map in the Go̅ model. The model with ϵ = 4.0 remains a fiber for more than 1 μs, but eventually starts to unravel from its ends, and its stacking interface also remains unclear [Figure 4A (model 4.0), Figure 4C (red)]. The model with ϵ = 4.5 generates a stable fiber where the monomers are in an ordered structure [Figure 4A (model 4.5), Figure 4C (purple)]. Models with ϵ = 5.0 and ϵ = 5.5 also produce a stable fiber, but force it into a strong twist [Figure 4A (models 5.0 and 5.5), Figure 4C (brown and pink)]. Based on the visual inspection of the results and the amount of conserved backbone contacts, we concluded that the model ϵ = 4.5 is the best choice for performing self-assembly simulations.

#### Self-Assembly

We studied the self-assembly of eight monomers over a period of 10 μs with the model 4.5 [Figure 4B,C (gray)]. In the initial situation, the monomers were separated from each other in the horseshoe conformation, but quickly opened to the extended conformation. During the first 200 ns, they began to aggregate, and in about 1 μs, four chains arranged in a horseshoe shape, forming a fiber precursor. In roughly 4 μs, the rest of the chains also settled into a horseshoe shape, however, forming their own fiber. The two fibers were in contact by interlocking hydrophobic interactions (Figure 4B). The self-assembly was repeated two more times, and in both cases the corresponding formation of a fiber precursor in the shape of a horseshoe was observed (see Figure S7).

## Conclusions

One of the biggest difficulties in coarse-grained simulations of proteins is the realistic description of their conformational changes. Even such central processes as protein folding, unfolding and self-assembly of proteins are processes whose description by CG models such as Martini 3 is nontrivial. The Go̅ model is one way to address this challenge. In this paper, we have presented a novel approach, which is particularly useful for studies of phenomena such as oligomerization and aggregation, where a large number of proteins interact simultaneously, and whose computational study requires massive parallelization. We have illustrated the advantages and limitations of this technique quite comprehensively and compared the quality of the Go̅ model implementations against the commonly used approach based on ENs.

### Oligomer Stability

To test oligomer formation and stability, we studied aquaporin, insulin, and amyloid-β proteins. The use of intramolecular ENs showed that only aquaporin (a membrane protein) forms a stable oligomeric structure in Martini 3. The structures of both insulin and amyloid-β quickly fall apart without additional support. By adding the properly parametrized Go̅-like bonds, it was possible to stabilize the structures of these protein complexes, and the internal fluctuations of the protein structures could be tuned to match the behavior of the atomistic models.

The intramolecular Go̅-potential (ϵ_intra_) of 15 kJ mol^–1^ established in previous studies is a reasonable starting value for all three protein systems, producing behavior that differs little from the results of atomic-level simulation models. We show with our insulin dimer stability assay (Figure 2) that a stable tertiary structure enables a stable interface, and vice versa. This reciprocal effect on the oligomer RMSF between tertiary and quaternary stability was not observed for the aquaporin tetramer (Figure 1). Therefore, a stronger ϵ_intra_ promotes more stable oligomerization in most cases (see Figure 2A,D). Such a high bias does come at a cost of lost chemical specificity, which is discussed in the following paragraph.

The results for amyloid-β show that a stable protein–protein interface can also stabilize the tertiary structure. The intramolecular Go̅ model of amyloid-β allows sampling of both extended and horseshoe conformations. An extended conformation is preferred for monomers in solution, whereby a horseshoe is dominantly formed as the fibers grow. This prion behavior is known to occur for amyloid-β and is thought to play a role in the progression of Alzheimer’s disease. As a continuation of this research, it would be fascinating to investigate the role of cell membranes, especially in the early stages of the formation of amyloid fibers.

Unlike the other proteins studied, aquaporin appears to be stable regardless of the applied Go̅ potentials, suggesting that the aquaporin complex is kept stable by a phenomenon different from the cases of insulin and amyloid-β. A possible explanation for the exceptional stability of aquaporin is that the mismatch between the protein’s hydrophobic region and membrane thickness is the main driving force for oligomerization. Such a driving force is lacking in water-soluble proteins.

### Chemical Specificity

Based on the results, the intermolecular Go̅ potential (ϵ_inter_) should be chosen to a rather high level for the insulin dimer (15 kJ mol^–1^), so that the quaternary structure can be stabilized and correspond as accurately as possible to the simulated atomic-level models. The problem is that such a high Go̅ potential causes oligomerization to become independent of the chemical environment. This is shown in Figure 3B,D, where any value of ϵ_inter_ greater than 10 kJ mol^–1^ results in a stable dimer. Such high Go̅ potentials cause the interface to behave like a glass, which means that self-assembly becomes difficult because the protein aggregate is trapped in suboptimal local minima and does not sample the global minimum to form interactions that occur in native structures, which are nevertheless observed experimentally.

We have shown that lower values of ϵ_inter_ (between 4 and 10 kJ mol^–1^) produce weaker protein complexes and allow the qualitative influence of the chemical environment. The goal would then be to use the lowest possible Go̅ potential resulting in a Martini 3 model with as little deviation as possible, while still forming the oligomer at the target concentrations. Keep in mind that adding Go̅ potentials with values below 4 kJ mol^–1^ for the intermolecular interactions does not make sense since the native backbone–backbone interaction in Martini 3 is 4.06 kJ/mol^–1^ or less, depending on the amino acid and the size of the backbone bead used.

If the binding and unbinding energies of proteins are known either from experiments or atomistic simulations, ϵ_inter_ could be tuned to accurately reproduce the correct binding energies. In this situation, it would even be possible to move away from uniform Go̅-model interaction potentials and tune them separately for each residue, although this would require very high-quality data, which are often unavailable or difficult to sample from atomistic simulations or experiments.

### Large Numbers of Protein Monomers

We implemented the “Snake oil” method in our model to allow simulation conditions for Martini 3 that contain multiple copies of the protein affected by the Go̅ potential. By adding four virtual beads per backbone, we have full control over the intramolecular and intermolecular bias potentials, including cancellation of the original potentials, by defining only intramolecular and intermolecular contacts on a single molecule. This differs from the previous methods where protein cross-interactions are defined between molecules, meaning that each cross-interaction must be explicitly defined between each instance of the molecule. In our method, this need does not exist; the number of proteins can be safely changed in the GROMACS topology file without unexpected side effects. This was demonstrated by the amyloid-β studies, where the same ITP files were used for a preformed fiber (27 copies) and self-assembly (8 copies). This also means that the order in which the monomers reside in the fiber is not relevant for the biasing potential.

The downside of the “Snake oil” approach is that two virtual sites must be added to each C_α_ if it is associated with an intra- or intermolecular Go̅ potential. This means that a C_α_ bead can contain up to 4 additional virtual sites. The exact effect on performance is difficult to determine, but in our tests it proved to be unnoticeable. This is probably due to the relatively low number of C_α_ beads in our test systems. In more diverse protein systems, the effect may be more significant. But even under such conditions, adding virtual sites should only affect the computational cost of the simulation, since increase in particle/site number can be counteracted by increasing parallelization. Therefore, the maximum simulation time or wall clock time is not significantly affected by the total number of added virtual sites.

### Outlook

During review we took notice of two preprint manuscripts on Martini plus Go̅ models which we feel should be mentioned. The work of de Souza et al. shows how Go̅ models can be added natively to Martinize2.^27^ Pedersen et al. focus on how to calculate the contact map from a more bottom up understanding.^42^ Both the ease of use and the hydrogen-bond based contact map are approaches we would like to merge with our snake-oil method in the future.

The application of Go̅-like potentials to support the tertiary and quaternary structures of CG proteins paves the way for simulation studies of protein folding/unfolding as well as simulations of self-assembly processes in larger protein complexes, such as formation of viral protein capsids.

## Supporting Information Available

Implementation details of the Go̅-like potentials and “Snake oil” model (text, figures); RMSF value comparison for the aquaporin tetramer (plot); RMSF, rmsd, and COM values for the insulin dimer (plots); results of the additional self-assembly repeats of the amyloid-β fiber simulations (figure); source code of the used scripts (PDF)
