# Supplementary material for: Martini 3 OliGo̅mers: A Scalable Approach for Multimers and Fibrils in GROMACS
Source: J Chem Theory Comput. 2024 Aug 27;20(17):7635–45. doi: 10.1021/acs.jctc.4c00677 (PMC11391574; doi:10.1021/acs.jctc.4c00677)
Supplement: Supplementary file 1 — ct4c00677_si_001.pdf [file ct4c00677_si_001.pdf]

Supporting Information for Martini 3 OliGōmers: A Scalable Approach for Multimers and Fibrils in GROMACS

Ksenia Korshunova,*,†,‡ Julius Kiuru,†,‡ Juho Liekkinen,† Giray Enkavi,† Ilpo Vattulainen,† and Bart M H Bruininks*,†

†*Department of Physics, University of Helsinki, FI-00014 Helsinki, Finland*

‡*Contributed equally to this work.*

E-mail: ksenia.korshunova@helsinki.fi; bartbruininks@gmail.com

Gō-bonds implementation

The Martini-Gō scheme is realized by applying Lennard-Jones (LJ) potentials to a subset of non-local pairwise contacts (Gō-like contacts) of the coarse grained protein structure.[1] The LJ potential is defined as

$$U_{LJ}(r_{ij}) = 4\epsilon_{ij}\left[\left(\frac{\sigma_{ij}}{r_{ij}}\right)^{12} - \left(\frac{\sigma_{ij}}{r_{ij}}\right)^{6}\right] , \quad (1)$$

where ϵ_{ij} is the depth of the potential energy well determining the strength of the attractive i-j interaction, and σ_{ij} is the distance at which the interaction energy of the particle pair i-j becomes 0. The Gō-like contacts are formed based on the contact map defined by the native structure of the protein. Here, the OV+rCSU type of contact map was used.[2]

The workflow of the present Martini-Gō scheme is as follows (Figure S1). Firstly, the atomistic protein complex structure in its native conformation (reference structure) is coarse

grained using the martinize2 script.[3,4] The reference structure is also used for creating the contact map via the rCSU server http://info.ifpan.edu.pl/ rcsu/rcsu/index.html.[2] The resulting CG structure and the contact map are then used as input for the in-house version of the create_goVirt.py script (originally created by Poma et al.[1]). The script requires the user to define the start and end indices for the segments (chains) of the protein complex. This has the advantage of avoiding the ambiguity in the definition of a separate protein chain (e.g. if the definition is based on the distance condition alone, or when the system contains chains connected by disulfide bonds) and instead allowing the user full control over the system definition. Based on the provided contact map and the list of the protein chains, the script assigns i-j pairs located within the same chain ϵ_{intra}, whereas i-j pairs with CG beads belonging to different chains are assigned the value of ϵ_{inter}. The values of ϵ_{intra} and ϵ_{inter} are user-defined. This emulates the tertiary (within chain) and quaternary (between chains) protein structure tiers. Thus, within a single chain, Gō-like contacts are defined with "intramolecular" potentials, whereas separate chains interact via "intermolecular" potentials. The intermolecular potentials are weaker than the intramolecular ones by default, allowing for more transient interactions between chains, compared to the interactions inside a chain.

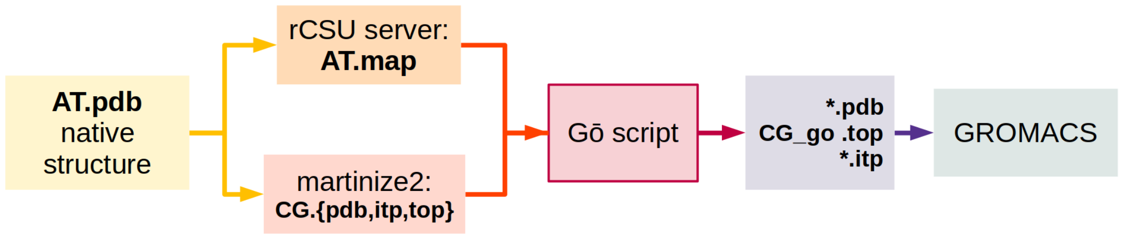

Figure S1: General workflow of a Martini-Gō simulation.

In this implementation, the stabilizing LJ potentials are applied to the virtual sites (VS) located at the positions of the backbone (BB) CG particles of the structure. The use of the VS is required due to the implementation of the non-bonded interactions in GROMACS: only one pairiwise interaction of a certain type (here: LJ) can be assigned to a pair of particles.

Finally, the script produces a set of GROMACS-compatible files, including the structure (`.pdb`) and topology (`.itp, .top`) files.

"Snake oil" model implementation

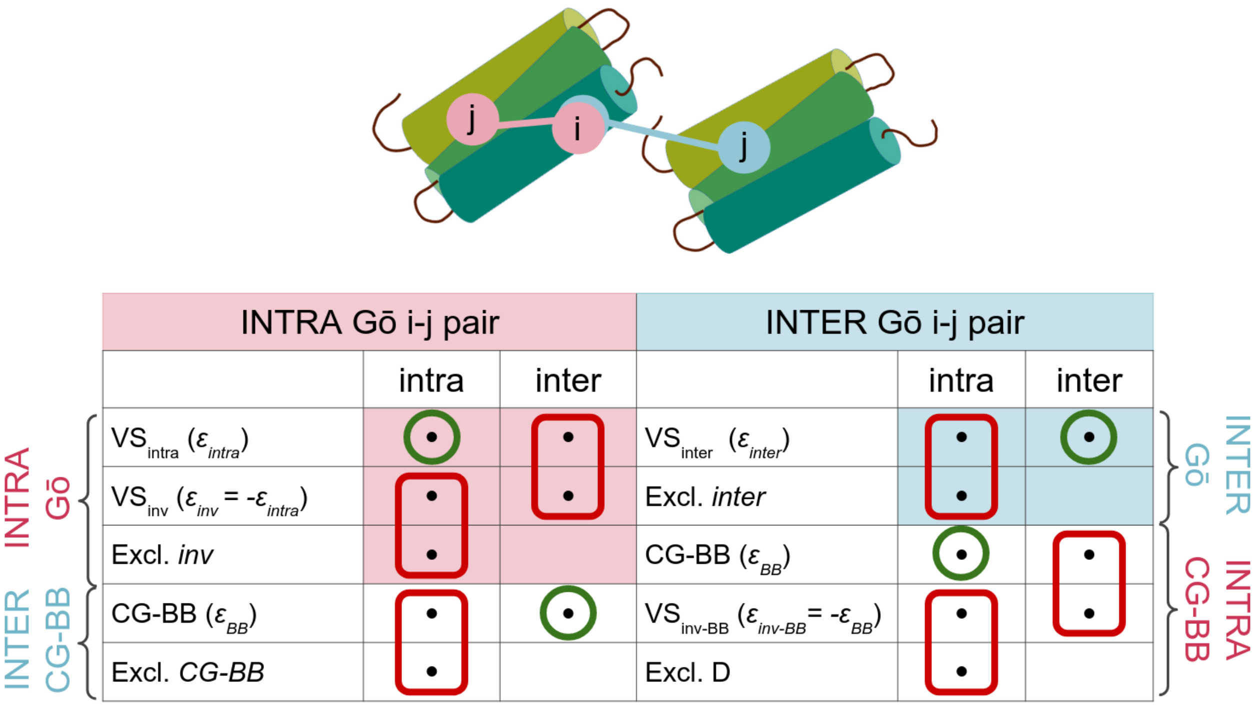

Figure S2: Graphic representation of the multilayer virtual site (VS) scheme illustrated on the example of one intramolecular (pink) and one intermolecular (light blue) i-j pair interaction between two copies of a protein chain. Each unique interaction (VS, Martini bead, exclusion) is represented as a row in the table, with dots in the cells where this interaction applies. The red squares indicate interaction cancellation, the green circles highlight the effective remaining interaction resulting from the layered setup. It is important to know that exclusions are only active in GROMACS for intramolecular interactions.

An alternative script version was developed in-house for the specific application to homopolymer systems. Similar to the version used for the aquaporin and insulin dimer test cases, the "snake oil" script takes an assembled reference system as input, analyzes the contact map, then separates the contacts into the intra- and intermolecular types. In the specific case of the homopolymers, however, it is possible to reduce the size and complexity of the resulting output topology via an additional processing step. Here, both the intra- and intermolecular

Gō-like contacts are mapped onto a single chain from the original input assembly via the modulo operation. The output of the script is a set of topology files and a coordinate file describing one chain. This approach reduces the size of the output and allows for construction of arbitrarily large systems by simply adding more copies of the desired complex (single chain or combinations) – a feature which was not present in the Poma,[1] de Souza,[5] and Thallmair[6] implementations.

In a system with multiple copies of the same chain, the following case is likely to occur: there exists both an intramolecular i-j pair and an intermolecular pair between bead i of chain A and bead j of chain B* (see the schematic representation in Figure S2). To correctly implement the separation between the intra- and intermolecular Gō bonds within the framework of GROMACS[7–9] single chain topology, a multi-layer virtual sites (VS) scheme was developed.

The goal of the clear separation between the intra- and intermolecular pair sets is achieved by using combinations of the standard $V(\epsilon)$ and inverted $V(\epsilon_{inv})$ LJ interactions ($\epsilon_{inv} = -\epsilon$)†, as well as exclusions. This can be illustrated using the intermolecular pair interaction as an example (Figure S2, "INTER Gō i-j pair" column). Each row in the column represents a unique type of GROMACS-implemented interaction: LJ interactions between virtual sites ("VS"), LJ interactions between backbone beads onto which the VSs are mapped ("CG-BB"), and exclusions ("Excl."). A non-bonded interaction between a pair of particles (or virtual sites) is applied indiscriminately both within a single chain and between chain copies in GROMACS. In the table, this is indicated by the black dots in both "intra" and "inter" sub-columns. GROMACS exclusions, on the other hand, can be applied only within a chain, which in this context is defined as a unique `[ moleculetype ]` (see GROMACS documentation[10] for more details), therefore, exclusions only have dots in the "intra" column. To define a purely intermolecular interaction for a given i-j pair, a standard VS-VS interaction

*After the modulo operation, bead $j + k * N$ where N is the total chain length and k is a positive integer maps onto bead j of the output chain.

†To avoid numerical instabilities, the absolute value of ϵ_{inv} deviates from ϵ by 0.00001 kJ/mol.

is first defined (VS_{inter}), then an exclusion is applied, which cancels all interaction of this type within the chain (indicated by the red square around the cancelled pair in Figure S2), leaving only the intermolecular *i-j* interactions (circled in green). Additionally, a correction for the underlying Martini bead pair is added to ensure that, while the intermolecular Martini bead-bead interaction is cancelled (to not interfere with the $VS_{i\text{-}j}$ pair), the intramolecular bead-bead interaction is intact. A similar principle is used to create exclusively intramolecular interactions.

Aquaporin tetramer

To ensure that the choice of the trajectory interval did not cause deviations in the RMSF values of the CG structure, we tested four additional 250 ns long intervals (1500-1750 ns, 2500-2750 ns, 3500-3750 ns, 4500-4750 ns) in comparison with the interval used in the manuscript. The values show a nearly identical overlap, confirming that the results and conclusions do not depend on the choice of the time window.

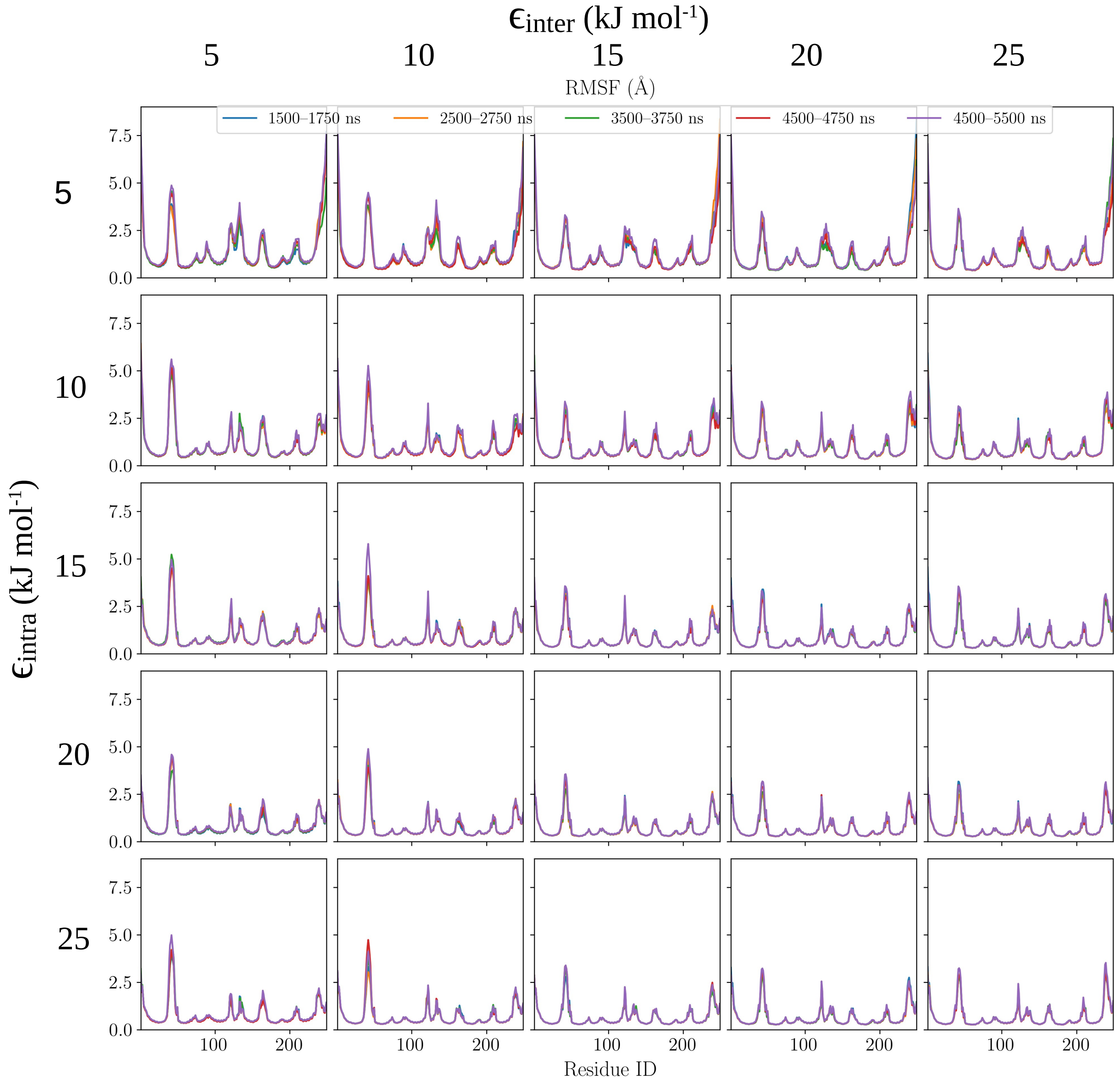

Figure S3: Comparison of RMSF values of CG-G$\bar{o}$ models of aquaporin for different intervals of the trajectory: four 250 ns intervals and the final 20% of the production run. The RMSF values are calculated as averages over all three repeats and four monomers of the aquaporin tetramer. The grid is in the same order as in Figure 1C in the manuscript.

Insulin dimer

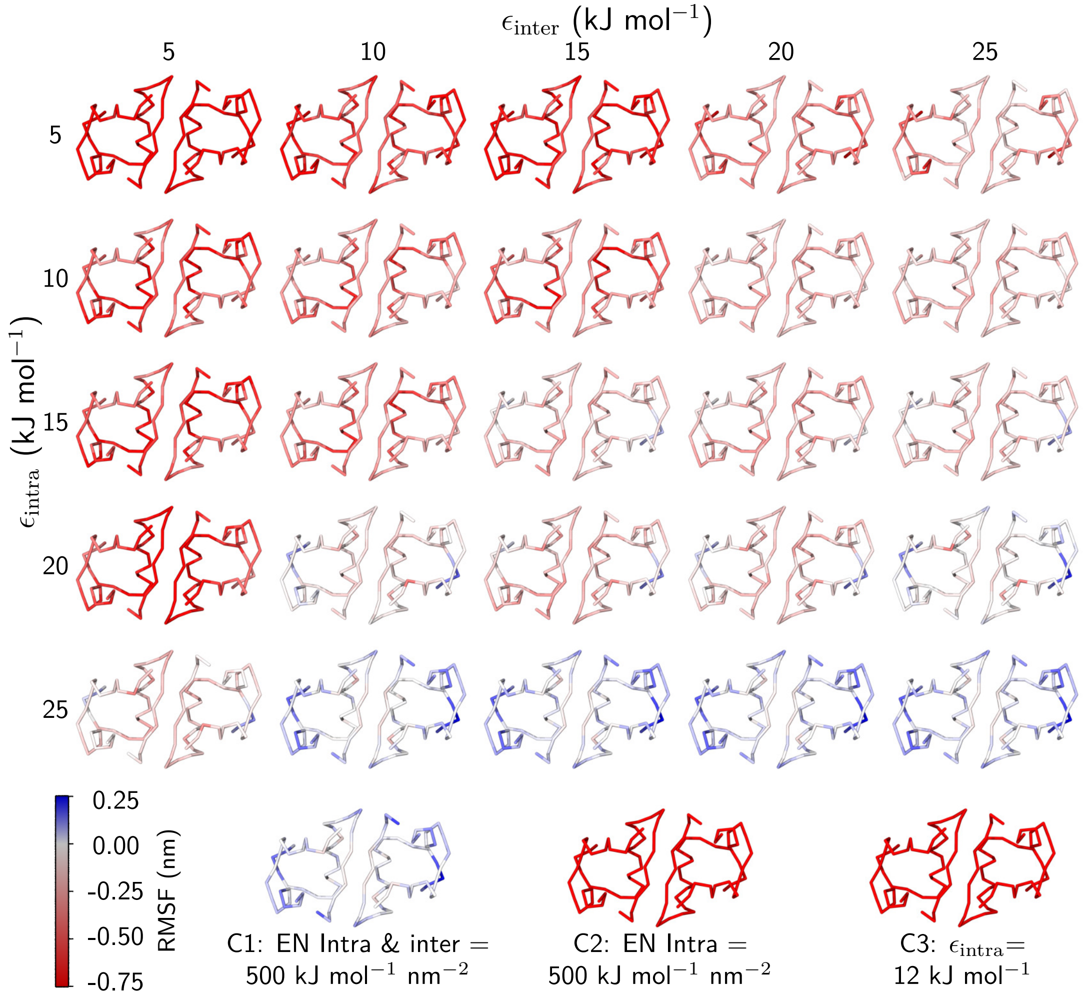

Figure S4: RMSF values calculated for the insulin (5BTS) dimer. RMSF for CG values are shown as the difference between the atomistic and CG RMSF. Negative values indicate that the CG structure is less stable and/or more flexible than the atomistic version while blue coloring suggests that the CG structure is more rigid. Dimer analysed as two separate molecules.

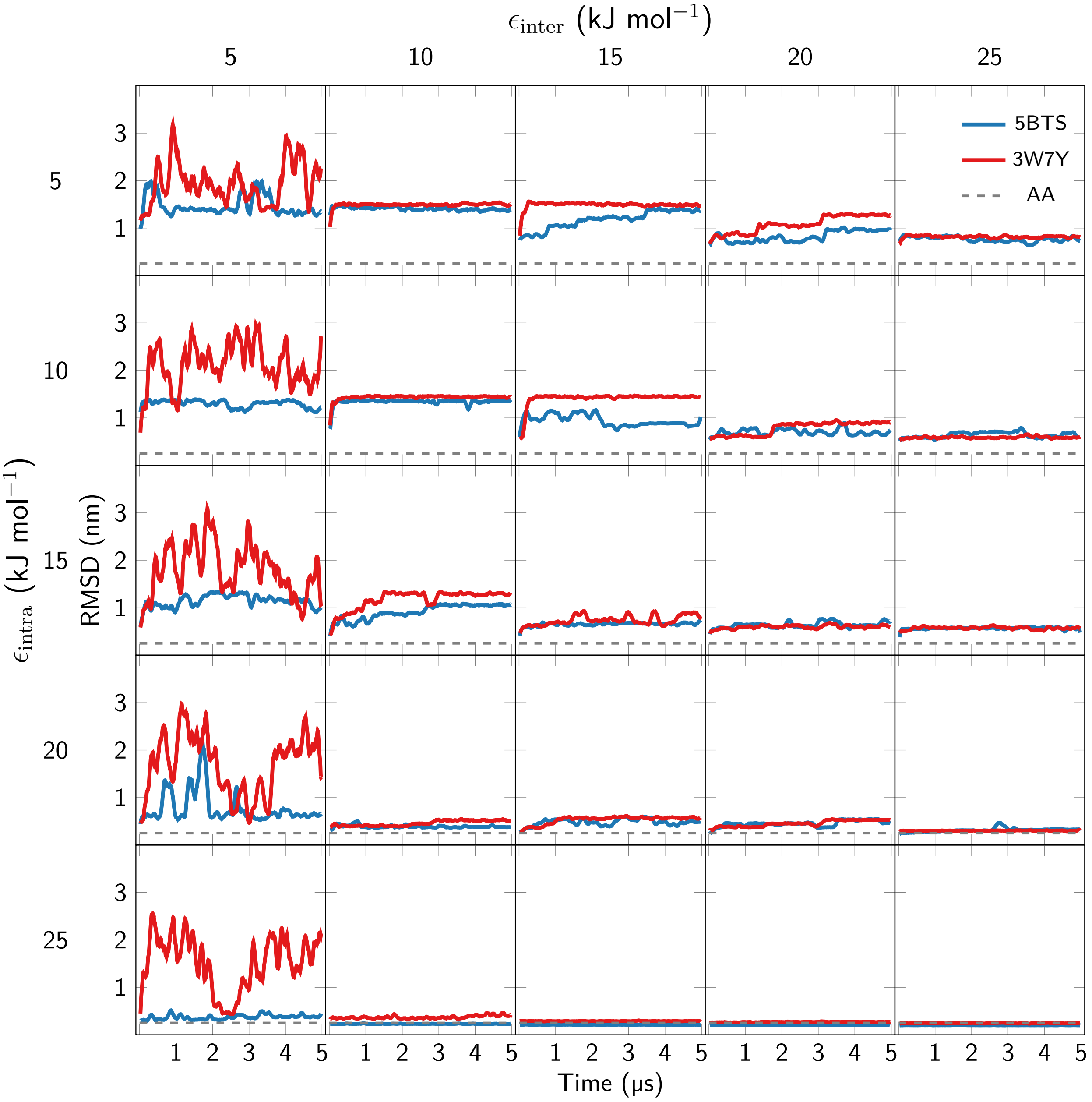

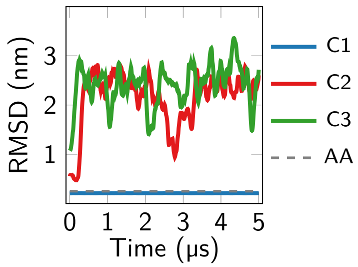

Figure S5: RMSD of 5BTS and 3W7Y dimers in the simulations with different ϵ_{inter} and ϵ_{intra} values. The dashed line indicates the average RMSD of the insulin dimer in the 500 ns atomistic simulations. The control systems are C1: EN intra & inter = 500 kJ mol^{-1} nm^{-2}, C2: EN intra = 500 kJ mol^{-1} nm^{-2}, and C3: ϵ_{intra} = 12 kJ mol^{-1}.

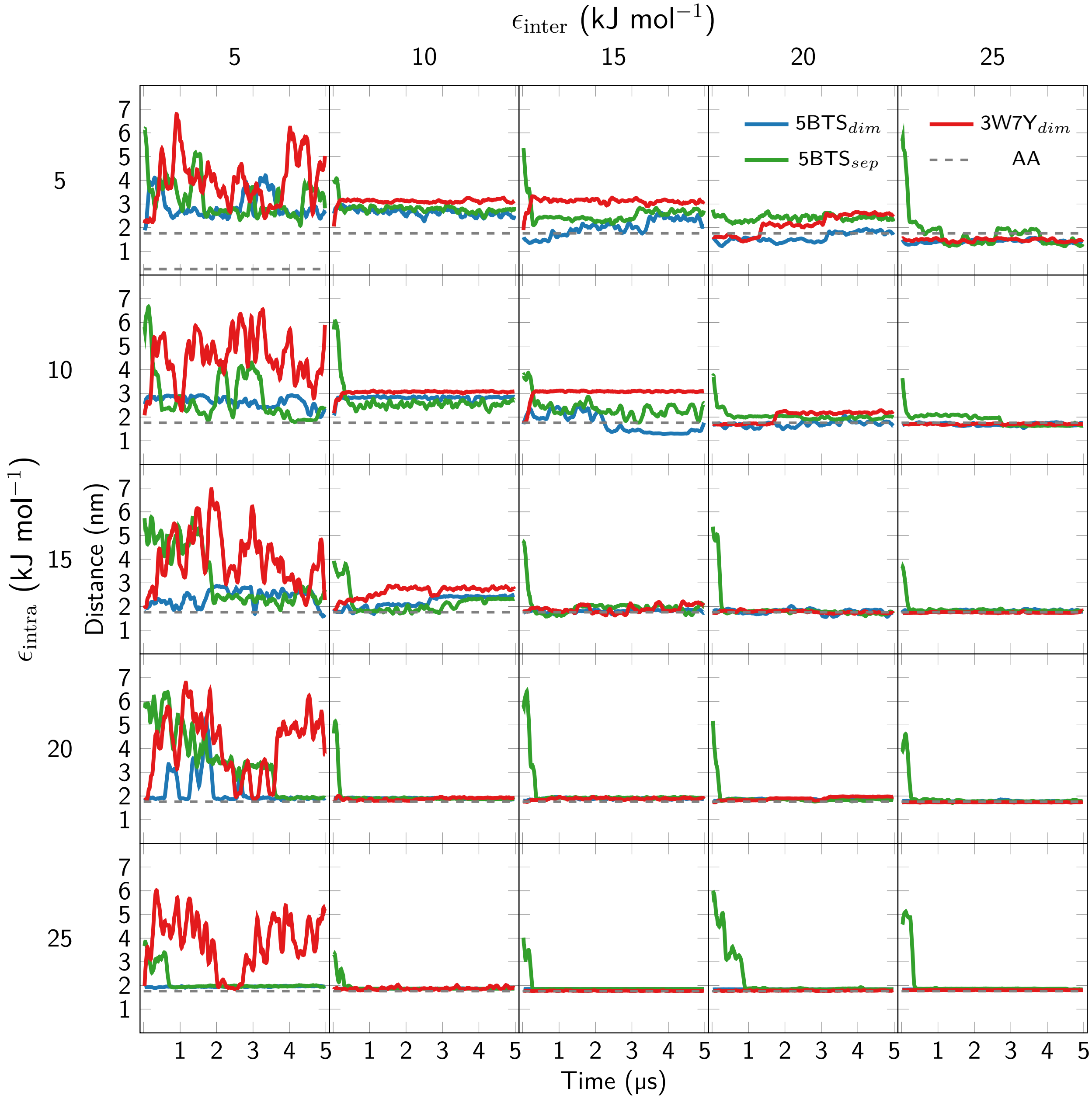

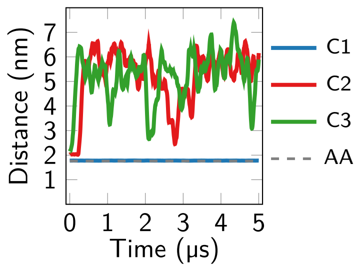

Figure S6: The center of mass (COM) distance between the 5BTS and 3W7Y insulin molecules in the simulations with different ϵ_{inter} and ϵ_{intra} values. The dashed line indicates the average COM distance of the insulin dimer in the 500 ns atomistic simulations. The control systems are C1: EN intra & inter = 500 kJ mol^{-1} nm^{-2}, C2: EN intra = 500 kJ mol^{-1} nm^{-2}, and C3: ϵ_{intra} = 12 kJ mol^{-1}.

Amyloid-β fiber

Self-assembly repeats

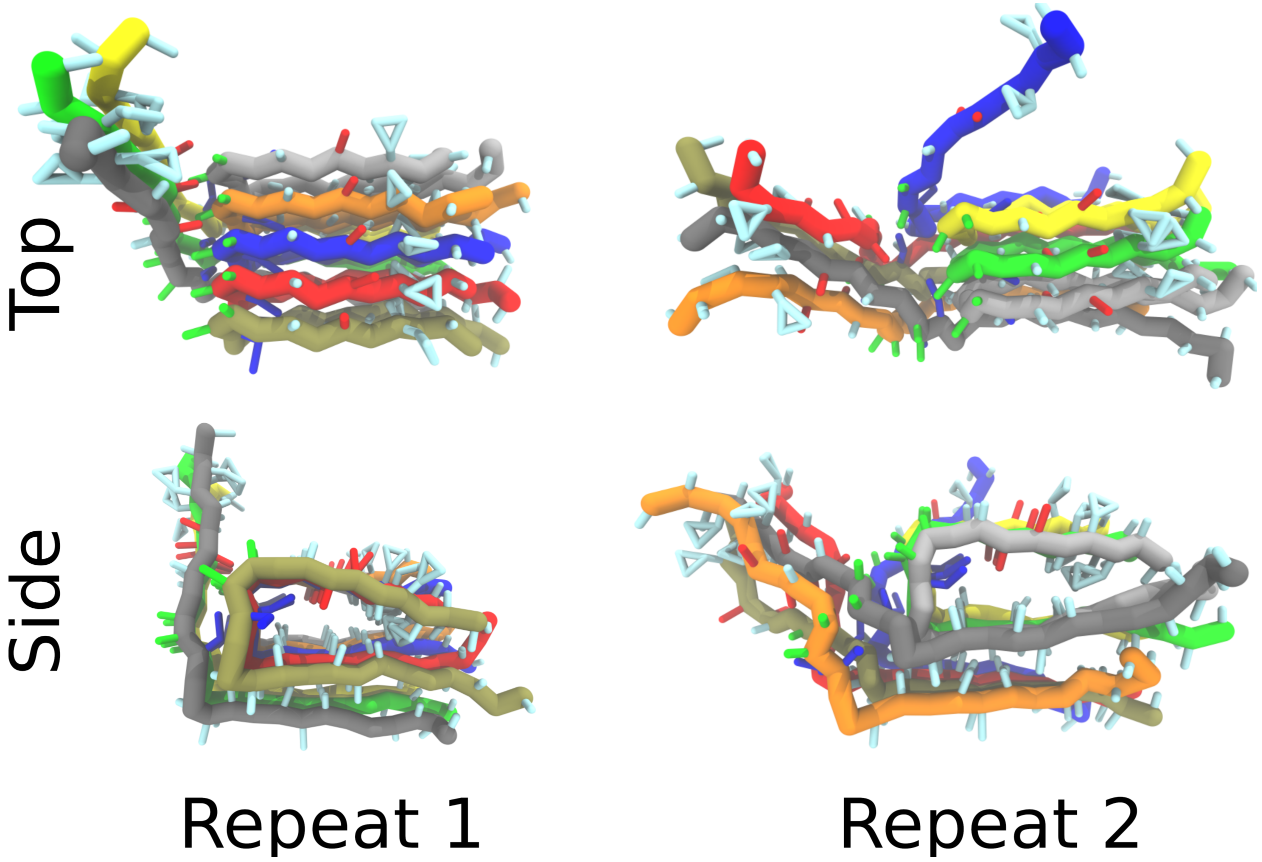

Figure S7: The top and side views of two additional repeats of the 10 μs amyloid-β self-assembly (8 peptides). In both repeats a single fiber seed is formed. The remaining chains which are not part of the structured fiber are wrapping around the seed, mainly by alignment of hydrophobic residues. For repeat 1 the seed consists of 5 chains, repeat 2 has a structured seed of 3 chains. Chains are annotated with unique colours and the side chains are colored by residue type. Red (negative), deep-blue (positive), green (polar), sky-blue (aliphatic).

Calculation of relative contacts

The relative contacts for the self-assembly of the amyloid-β fiber were calculated using the following script.

In [1]:
```python
import MDAnalysis as mda
import numpy as np
import matplotlib.pyplot as plt
from collections import Counter
```

In [2]:
```python
# The first frame is the preassembled fiber
#  (contacts based on alignment of strand B-C
#  of 2beg amyloid beta)
universe_ref = mda.Universe('../fiber_go_center.pdb',
                            '../fiber_go_center.pdb')
selection_ref = universe_ref.select_atoms('name BB')
#universe_data = mda.Universe('../fiber_go_center.pdb',
#                              '../fiber_go_center.pdb')
#selection_data = universe_ref.select_atoms('name BB')
universe_data = mda.Universe('../fiber_go_center.pdb',
                             '../whole.xtc')
# Making a subselection is possible
selection_data = universe_data.select_atoms('name BB')
#selection_data = universe_data.select_atoms(
#    'name BB and chainID B H F D')
cutoff = 6 # Angstrom
intra = False
query_chainID = 'D' # This is used as the reference chain
```

In [3]:
```python
def create_chain_starting_indices_dict(universe):
    """
    Returns a dict with the first index for every chain.
    """
    unique_chains = np.unique(universe.atoms.chainIDs)
    chain_starting_indices = [universe.select_atoms(
        f'chainID {id}')[0].ix for id in unique_chains]
    chain_starting_indices_dict = dict(
        zip(unique_chains, chain_starting_indices))
    return chain_starting_indices_dict
```

In [4]:
```python
def atom_local_env(query_atom, cutoff, selection,
                   chain_starting_indices_dict,
                   intra=False):
    """
    Returns a dict of the local relative environment
    count around the atom within given cutoff (A) of
    beads in the selection.
    """
    atom_chain_id = query_atom.chainID
    index_environment = selection.select_atoms(
        f'around {cutoff} index {query_atom.ix}')
    index_environment_indices = index_environment.ix
    index_environment_chainIDs = index_environment.chainIDs
    #print(index_environment.names)
    #print(index_environment.chainIDs)
    index_environment_relative_indices = []
    for index, chainID in zip(
        index_environment_indices,
```

```
        index_environment_chainIDs):
        #print(chainID, atom_chain_id)
        if not intra and chainID == atom_chain_id:
            continue
        #print(chainID)
        index_environment_relative_indices.append(
            index - chain_starting_indices_dict[chainID])
    output = dict(zip(*np.unique(
        index_environment_relative_indices,
        return_counts=True)))
    return output
```

In [5]:
```
def chain_local_env(chain, cutoff, selection,
                    chain_starting_indices_dict,
                    intra=False):
    """
    Returns a dict of the local relative environment
    count around the chain within given cutoff (A) of
    beads in the selection.
    """
    all_envs = []
    for atom in chain.atoms:
        all_envs.append(
            atom_local_env(atom, cutoff, selection,
                           chain_starting_indices_dict,
                           intra=intra))
    return all_envs
```

In [6]:
```
def diff_contacts(contactsA, contactsB, return_count=True):
    """
    Returns the difference in contacts between the two contact dicts.
    """
    c1, c2 = Counter(contactsA), Counter(contactsB)
    diff = dict((c1-c2) + (c2-c1))
    if return_count is True:
        return sum(diff.values())
    else:
        return diff
```

In [7]:
```
# Obtain the ids for the first index in the chain for relative indexing.
chain_starting_indices_dict = create_chain_starting_indices_dict(
    universe_ref)
```

In [8]:
```
# Set the baseline local environment from taking a central chain
#  in the fiber.
query_chain = universe_ref.select_atoms(
    f'chainID {query_chainID} and name BB')
chain_ref_contacts = chain_local_env(
    query_chain, cutoff, selection_ref,
    chain_starting_indices_dict, intra=intra
)
amount_of_data_selection_chains = len(
    np.unique(selection_data.atoms.chainIDs))
total_ref = [sum(ref_contacts.values())
             for ref_contacts in chain_ref_contacts]
```

```
total_ref = sum(total_ref) * amount_of_data_selection_chains
print(f'There are {total_ref} total perfect fiber contacts.')
```

```
There are 864 total perfect fiber contacts.
```

In [9]:

```
chain_starting_indices_dict = create_chain_starting_indices_dict(
    universe_data)
all_diff = []
time_stamps = []
step_size = 1
for idx, frame in enumerate(universe_data.trajectory[0:10001:step_size]):
    time_stamps.append(step_size*idx)
    print(f'\rProcessing frame {frame}.', end='')
    frame_diff = 0
    for query_chainID in chain_starting_indices_dict.keys():
    #for query_chainID in ['B', 'H', 'F', 'D']:
        query_chain = universe_data.select_atoms(
            f'chainID {query_chainID} and name BB')
        #print(query_chain.chainIDs[0])
        temp_contacts = chain_local_env(
            query_chain, cutoff, selection_data,
            chain_starting_indices_dict, intra=intra)
        chain_diff = 0
        for idx in range(0, len(chain_ref_contacts)):
            chain_diff += diff_contacts(
                chain_ref_contacts[idx],
                temp_contacts[idx],
                return_count=True)
        frame_diff += chain_diff
    all_diff.append(frame_diff)

all_diff = np.array(all_diff)
```

```
Processing frame < Timestep 10000 with unit cell dimensions [115.17265 115.1
7265 115.17265  90.       90.       90.     ] >. ] >.
```

In [10]:

```
# create n shifts and std and average them manually.
naverage = 100
stacked_arrays = np.zeros((naverage,*all_diff.shape))
stacked_arrays[0] = all_diff
for idx in range(1,naverage):
    stacked_arrays[idx, 0:-idx] = all_diff[idx:]
mean_array = np.mean(stacked_arrays,axis=0)[:-naverage]
std_array = np.std(stacked_arrays,axis=0)[:-naverage]
print(mean_array.shape)
```

```
(9901,)
```

In [11]:

```
plt_mean_array = ((total_ref-mean_array)/total_ref)*100
# Notmalized std
plt_std_array = std_array * (100/total_ref)
print(plt_mean_array.shape)
print(len(time_stamps[naverage//2:-naverage//2]))
```

```
(9901,)
9901
```

In [12]:
```
plt.plot(time_stamps[naverage//2:-naverage//2],
         plt_mean_array, label='self assebly')
plt.fill_between(time_stamps[naverage//2:-naverage//2],
                 plt_mean_array - plt_std_array,
                 plt_mean_array + plt_std_array,
                 alpha=0.35)
plt.xlim(0, len(time_stamps)*step_size)
plt.ylim(0, 100)
plt.xlabel('time (ns)')
plt.ylabel('relative contacts (%)')
plt.legend()
plt.savefig('relative_contacts_per_atom.png', dpi=600)
plt.savefig('relative_contacts_per_atom.svg')
plt.show()
```

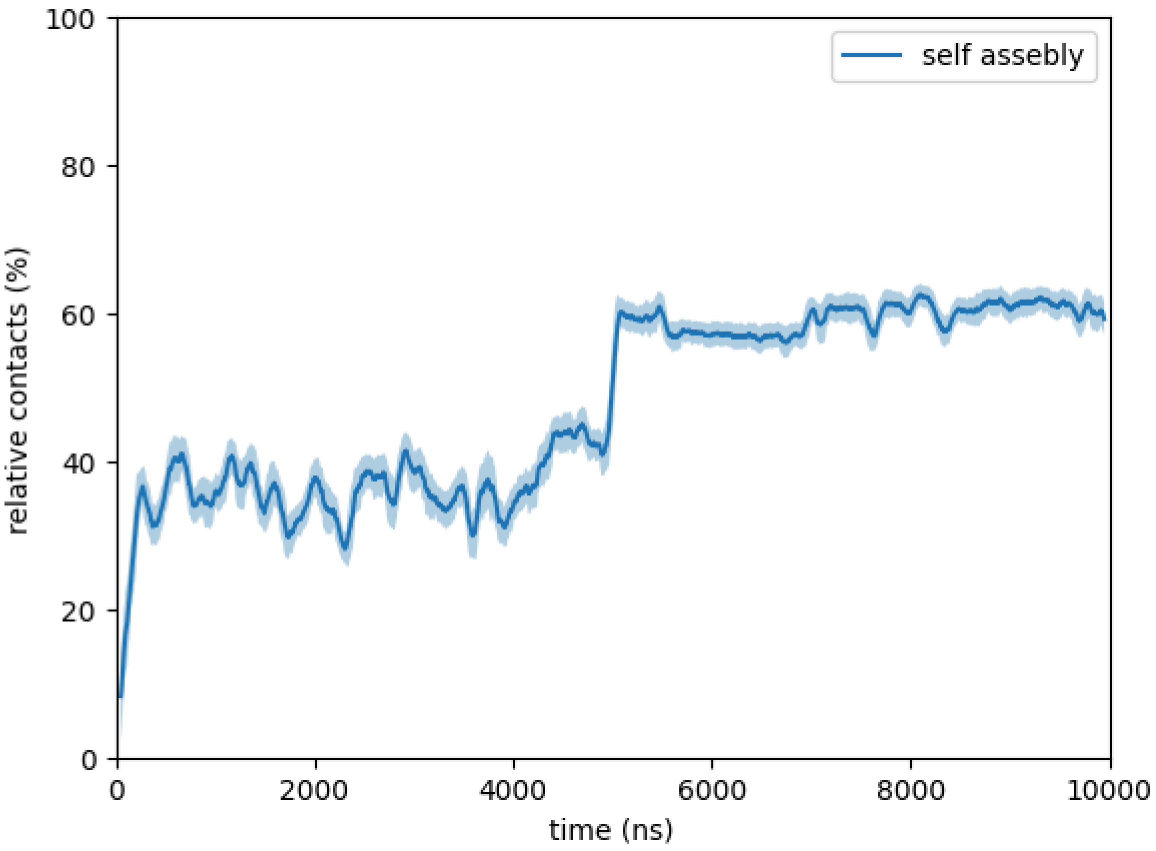

In [13]:
```
output_data = np.array(all_diff)
np.save('relative_contacts_per_atom.npy', output_data)
```

Scripts

create_goVirt.py

create_goVirt.py script, from the 2021 Martini online workshop. The script is used to create the Gō bonds in the intramolecular Gō model.

```
#!/usr/bin/env python

import argparse
import subprocess
import numpy as np


def user_input():
    parser = argparse.ArgumentParser()
    parser.add_argument('-s', help='File containing the coarse-grained structure of the protein in pdb format.')
    parser.add_argument('-f', help='File containing the contact analysis of the (atomistic) protein structure'+
                        ' obtained from the webserver http://info.ifpan.edu.pl/~rcsu/rcsu/index.html.')
    parser.add_argument('--moltype', default='molecule_0', help='Molecule name used as prefix in your output file'+
                        ' names and the virtual bead names (default: molecule_0). If you will combine your Go-like'+
                        ' model with a coarse-grained protein generated with martinize2, you must use the same name'+
                        ' as specified with the --govs-moltype flag of martinize2!')
    parser.add_argument('--go_eps', type=float, default=9.414, help='Dissociation energy [kJ/mol] of the'+
                        ' Lennard-Jones potential used in the Go-like model (default: 9.414).')
    parser.add_argument('--cutoff_short', type=float, default=0.3, help='Lower cutoff distance [nm]: contacts with'+
                        ' a shorter distance than cutoff_short are not included in the Go-like interactions (default: 0.3).')
    parser.add_argument('--cutoff_long', type=float, default=1.1, help='Upper cutoff distance [nm]: contacts with'+
                        ' a longer distance than cutoff_long are not included in the Go-like interactions (default: 1.1).')
    parser.add_argument('--Natoms', type=int, help='Number of coarse-grained beads in the protein excluding the virtual Go beads.')
    args = parser.parse_args()
    return args

def get_settings():
    # names for temporary files:
    file_BB = 'BB.pdb'
    file_OV = 'OV.map'
    file_rCSU = 'rCSU.map'

    # some other rudimentary variables
    header_lines = 0
    seqDist = 4         # minimal distance in the sequence to add a elastic bond (ElNedyn=3 [Perriole2009]; Go=4 [Poma2017])
    cols = [5, 9, 10]   # colums of interest in the OV and rCSU contact map file
    missRes = 0         # number of missing residues at the beginning of pdb structure
                        # (this has to result in the correct residue number when added to "k_res" compared to the .pdb file)
    missAt = 0          # number of missing atoms at the beginning of pdb structure
                        # (this has to result in the correct atom number when added to "k_at" compared to the .itp file)
    c6c12 = 0           # if set to 1, the C6 and C12 term are expected in the .itp file; if set to 0, sigma and go_eps are used
    return file_BB, file_OV, file_rCSU, header_lines, seqDist, cols, missRes, missAt, c6c12

def read_data(struct_pdb, file_contacts, file_BB, file_OV, file_rCSU, header_lines, cols):
    # preparation of temporary files for reading
    subprocess.call("grep '1 [01] [01] [01]' " + file_contacts + " > " + file_OV, shell=True)
```

```
    subprocess.call("echo '' >> " + file_OV, shell=True)
    subprocess.call("grep '0 [01] [01] 1' " + file_contacts + " > " + file_rCSU, shell=True)
    subprocess.call("echo '' >> " + file_rCSU, shell=True)
    subprocess.call("grep 'BB' " + struct_pdb + " > " + file_BB, shell=True)
    subprocess.call("echo '' >> " + file_BB, shell=True)

    # read coarse-grained BB bead positions
    with open(file_BB,'r') as fid:
        dat = fid.readlines()
    dat = dat[header_lines:-1]
    print('Number of coarse-grained BB beads in your protein: ' + str(len(dat)))

    indBB = []
    nameAA = []
    for k in range(0, len(dat)):
        tmp = dat[k]
        tmp = tmp.split()
        indBB.append([ int(tmp[1]), float(tmp[6]), float(tmp[7]), float(tmp[8]) ])
        nameAA.append(tmp[3])
    indBB = np.array(indBB)

    # read OV contact map
    with open(file_OV,'r') as fid:
        dat = fid.readlines()
    dat = dat[header_lines:-1]
    print('Number of contacts read from your OV contact map file: ' + str(len(dat)))

    map_OVrCSU = []
    row = []
    for k in range(0, len(dat)):
        tmp = dat[k]
        tmp = tmp.replace('\t',' ')
        tmp = tmp.split()
        for l in cols:
            row.append(float(tmp[l]))
        map_OVrCSU.append(row)
        row = []

    # read rCSU contact map
    with open(file_rCSU,'r') as fid:
        dat = fid.readlines()
    dat = dat[header_lines:-1]
    print('Number of contacts read from your rCSU contact map file: ' + str(len(dat)))

    for k in range(0, len(dat)):
        tmp = dat[k]
        tmp = tmp.replace('\t',' ')
        tmp = tmp.split()
        for l in cols:
            row.append(float(tmp[l]))
        map_OVrCSU.append(row)
        row = []

    return indBB, nameAA, map_OVrCSU

def get_go(indBB, nameAA, map_OVrCSU, cutoff_short, cutoff_long, go_eps, seqDist, missRes):
```

```
    # calculate the distances based on the coordinates of the CG BB bead
    for k in range(0, len(map_OVrCSU)):
        dist_vec = indBB[ int(map_OVrCSU[k][1])-missRes-1 ,1:4] - indBB[ int(map_OVrCSU[k][0])-missRes-1 ,1:4]
        map_OVrCSU[k][2] = np.linalg.norm(dist_vec) /10     # [Ang] to [nm]

    pairs = []
    for k in range(0, len(map_OVrCSU)):
        if (map_OVrCSU[k][2] > cutoff_short) and (map_OVrCSU[k][2] < cutoff_long) and ( abs(map_OVrCSU[k][1]-map_OVrCSU[k][0]) >= seqDist ):
            # parameters for LJ potential
            sigma = map_OVrCSU[k][2] / 1.12246204830        # calc sigma for the LJ potential in [nm]
            Vii = 4.0 * pow(sigma,6) * go_eps
            Wii = 4.0 * pow(sigma,12) * go_eps
            pairs.append([indBB[ int(map_OVrCSU[k][0])-missRes-1 ,0], indBB[ int(map_OVrCSU[k][1])-missRes-1 ,0], Vii, Wii,
                          map_OVrCSU[k][0], map_OVrCSU[k][1], map_OVrCSU[k][2], sigma])
            Vii = []
            Wii = []
        elif map_OVrCSU[k][2] > cutoff_long:
            print('This contanct is excluded due to distance > cutoff_long: ' + str(map_OVrCSU[k]))
        elif map_OVrCSU[k][2] < cutoff_short:
            print('This contanct is excluded due to distance < cutoff_short: ' + str(map_OVrCSU[k]))
        elif abs(map_OVrCSU[k][1]-map_OVrCSU[k][0]) < 3:
            print('This contanct is excluded because the AA have less than ' + str(seqDist-1) + ' other AA between each other: '+
                  str(map_OVrCSU[k]))

    sym_pairs = []
    # count contacts only once; exclude asymmetric rCSU contacts (cf. doi 10.1063/1.4929599)
    for k in range(0, len(pairs)):
        if pairs[k][0] < pairs[k][1]:
            for l in range(k+1, len(pairs)):
                if (pairs[l][0] == pairs[k][1]) and (pairs[l][1] == pairs[k][0]):
                    sym_pairs.append(pairs[k])

    print ('- - - -')
    print ('These results exclude the contacts with distances higher than the cutoff_long (' + str(cutoff_long) + ' nm), shorter'+
           ' than the cutoff_short (' + str(cutoff_short) + ' nm), or where the AA have less than '+
           str(seqDist-1) + ' other AA between each other:')
    print ('Sum of symmetric (doubly counted) and asymmetric OV + rCSU contacts: ' + str(len(pairs)))
    print ('Only symmetric OV + rCSU contacts (singly counted):' + str(len(sym_pairs)))

    return sym_pairs

def write_files(file_pref, sym_pairs, missAt, indBB, missRes, Natoms, nameAA, go_eps, c6c12):
    # write the interaction table for the Go-like bonds
    with open(file_pref + '_go-table_VirtGoSites.itp','w') as f:
        f.write('; OV + symmetric rCSU contacts \n')
        if (c6c12 == 1):
            for k in range(0, len(sym_pairs)):
                # to write the LJ potential itp:
                s2print = " %s_%s  %s_%s    1  %.10f  %.10f  ;  %s  %s  %.3f \n" % (file_pref, str(int(sym_pairs[k][4])), file_pref,
                          str(int(sym_pairs[k][5])), sym_pairs[k][2], sym_pairs[k][3], str(int(sym_pairs[k][0]) +missAt),
                          str(int(sym_pairs[k][1]) +missAt), sym_pairs[k][6])
                          # atom index and residue index adapted due to missing residues
                f.write(s2print)
        else:
            for k in range(0, len(sym_pairs)):
                # to write the LJ potential itp:
```

```
                s2print = " %s_%s  %s_%s    1  %.10f  %.10f  ;  %s  %s  %.3f \n" % (file_pref, str(int(sym_pairs[k][4])), file_pref,
                          str(int(sym_pairs[k][5])), sym_pairs[k][7], go_eps, str(int(sym_pairs[k][0]) +missAt),
                          str(int(sym_pairs[k][1]) +missAt), sym_pairs[k][6])
                          # atom index and residue index adapted due to missing residues
                f.write(s2print)

    subprocess.call("echo '#include \"" + file_pref + "_go-table_VirtGoSites.itp\"' >> go-table_VirtGoSites.itp ", shell=True)

    # write supplementary file: BB virtual particle definitions for martini.itp
    with open(file_pref + '_BB-part-def_VirtGoSites.itp','w') as f:
        f.write('; protein BB virtual particles \n')
        for k in range(0, len(indBB)):
            s2print = "%s_%s 0.0 0.000 A 0.0 0.0 \n" % (file_pref, str(k+1 +missRes)) # residue index adapted due to missing residues
            f.write(s2print)
    subprocess.call("echo '#include \"" + file_pref + "_BB-part-def_VirtGoSites.itp\"' >> BB-part-def_VirtGoSites.itp ", shell=True)

    # write supplementary file: exclusions for protein.itp
    with open(file_pref + '_exclusions_VirtGoSites.itp','w') as f:
        f.write(';[ exclusions ] \n')
        f.write('; OV + symmetric rCSU contacts \n')
        for k in range(0, len(sym_pairs)):
            s2print = " %s  %s  \t ;  %s  %s \n" % (str(int(sym_pairs[k][0]) +missAt), str(int(sym_pairs[k][1]) +missAt),
                                                    str(int(sym_pairs[k][4])), str(int(sym_pairs[k][5])))
                                      # atom index and residue index adapted due to missing residues
            f.write(s2print)

    # write supplementary file: Go-like bonds as harmonic bonds for visulization of the protein
    with open(file_pref + '_go4view_harm.itp','w') as f:
        f.write('; Go bonds as harmonic bonds between the virtual particles: \n')
        f.write('; OV + symmetric rCSU contacts \n')
        for k in range(0, len(sym_pairs)):
            # to write the harmonic bonds itp:
            s2print = " %s  %s  1  %s  1250  ; %s_%s  %s_%s \n" % (str(int(sym_pairs[k][4] +Natoms)), str(int(sym_pairs[k][5] +Natoms)),
                      str(round(sym_pairs[k][6],3)), file_pref, str(int(sym_pairs[k][4])), file_pref,
                      str(int(sym_pairs[k][5])))
                      # the bonds are added between the virtual particles
            f.write(s2print)
        for k in range(0, len(indBB)):
            if (np.sum(np.array(sym_pairs)[:,4]==k+1) + np.sum(np.array(sym_pairs)[:,5]==k+1)) == 0:
                s2print = " %s  %s  1  1.  1     ; %s_%s  %s_%s --> added for vmd \n" % (str(int(k+1 +Natoms)), str(int(k +Natoms)),
                          file_pref, str(k+1), file_pref, str(k))
                f.write(s2print)

def main():
    args = user_input()
    file_BB, file_OV, file_rCSU, header_lines, seqDist, cols, missRes, missAt, c6c12 = get_settings()

    indBB, nameAA, map_OVrCSU = read_data(args.s, args.f, file_BB, file_OV, file_rCSU, header_lines, cols)

    sym_pairs = get_go(indBB, nameAA, map_OVrCSU, args.cutoff_short, args.cutoff_long, args.go_eps, seqDist, missRes)

    write_files(args.moltype, sym_pairs, missAt, indBB, missRes, args.Natoms, nameAA, args.go_eps, c6c12)
    print('All symmetric OV and rCSU contacts written! Have fun!')

if __name__ == '__main__':
    main()
```

Modified create_goVirt.py

The script is used to create the Gō bonds in the intramolecular and intermolecular Gō model. The go_eps input parameter has been split into two input parameters, go_eps_intra and go_eps_inter, to make it possible to give different values to the intramolecular and intermolecular potential well depths. Additionally, the option to give two new input parameters, chain_start and chain_end, which are lists of the residue IDs of the first and the last residues of each monomer, respectively, has been added. These lists are used in the get_go() and write_files() functions to differentiate between intramolecular and intermolecular contacts. The variable intra is used to keep track of whether the contact being processed is intramolecular or intermolecular. Otherwise, the script functions the same as the previous script.

```
#!/usr/bin/env python3
# -*- coding: utf-8 -*-

import argparse
import subprocess
import numpy as np

##############################################################################################################
#                                                                                                            #
# make sure the resids in the pdb file that is used to create the contact map are unique and in rising order    #
# make sure the atom numbers in the cg pdb are unique and in rising order                                    #
#                                                                                                            #
##############################################################################################################

def user_input():
    parser = argparse.ArgumentParser()
    parser.add_argument('-s', help='File containing the coarse-grained structure of the protein in pdb format.')
    parser.add_argument('-f', help='File containing the contact analysis of the (atomistic) protein structure'+
                        ' obtained from the webserver http://info.ifpan.edu.pl/~rcsu/rcsu/index.html.')
    parser.add_argument('--moltype', default='molecule_0', help='Molecule name used as prefix in your output file'+
                        ' names and the virtual bead names (default: molecule_0). If you will combine your Go-like'+
                        ' model with a coarse-grained protein generated with martinize2, you must use the same name'+
                        ' as specified with the --govs-moltype flag of martinize2!')
    parser.add_argument('--go_eps_intra', type=float, default=9.414, help='Dissociation energy [kJ/mol] of the'+
                        ' Lennard-Jones potential used in the Go-like model (default: 9.414).')
    parser.add_argument('--go_eps_inter', type=float, default=12.000, help='Dissociation energy [kJ/mol] of the'+
                        ' Lennard-Jones potential used in the Go-like model (default: 9.414).')
    parser.add_argument('--cutoff_short', type=float, default=0.3, help='Lower cutoff distance [nm]: contacts with'+
                        ' a shorter distance than cutoff_short are not included in the Go-like interactions (default: 0.3).')
    parser.add_argument('--cutoff_long', type=float, default=1.1, help='Upper cutoff distance [nm]: contacts with'+
                        ' a longer distance than cutoff_long are not included in the Go-like interactions (default: 1.1).')
    parser.add_argument('--Natoms', type=int, help='Number of coarse-grained beads in the protein excluding the virtual Go beads.')
    parser.add_argument('--missres', type=int, default=0, help='Number of missing residues at the beginning of the'+
                        ' atomistic pdb structure which is needed if the numbering of the coarse-grained structure starts at 1'+
                        ' (default: 0).')
```

```
    parser.add_argument('--chain_start', type=int, nargs='+', default=[1,250,499,748], help='Resids for the first'+
                        ' residues of chains corresponding to the resids in the contact analysis file'+
                        ' (default: 1 250 499 748). The resids in the pdb file that is used to create the'+
                        ' contact analysis should be unique and in rising order.')
    parser.add_argument('--chain_end', type=int, nargs='+', default=[249,498,747,996], help='Resids for the last'+
                        ' residues of chains corresponding to the resids in the contact analysis file'+
                        ' (default: 249 498 747 996). The resids in the pdb file that is used to create the'+
                        ' contact analysis should be unique and in rising order.')
    args = parser.parse_args()
    return args

def get_settings():
    # names for temporary files:
    file_BB = 'BB.pdb'
    file_OV = 'OV.map'
    file_rCSU = 'rCSU.map'

    # some other rudimentary variables
    header_lines = 0
    seqDist = 4         # minimal distance in the sequence to add a elastic bond (ElNedyn=3 [Perriole2009]; Go=4 [Poma2017])
    cols = [5, 9, 10]   # colums of interest in the OV and rCSU contact map file
    missAt = 0          # number of missing atoms at the beginning of pdb structure
                        # (this has to result in the correct atom number when added to "k_at" compared to the .itp file)
    c6c12 = 0           # if set to 1, the C6 and C12 term are expected in the .itp file; if set to 0, sigma and go_eps are used
    return file_BB, file_OV, file_rCSU, header_lines, seqDist, cols, missAt, c6c12

def read_data(struct_pdb, file_contacts, file_BB, file_OV, file_rCSU, header_lines, cols):
    # preparation of temporary files for reading
    subprocess.call("grep '1 [01] [01] [01]' " + file_contacts + " > " + file_OV, shell=True)
    subprocess.call("echo '' >> " + file_OV, shell=True)
    subprocess.call("grep '0 [01] [01] 1' " + file_contacts + " > " + file_rCSU, shell=True)
    subprocess.call("echo '' >> " + file_rCSU, shell=True)
    subprocess.call("grep 'BB' " + struct_pdb + " > " + file_BB, shell=True)
    subprocess.call("echo '' >> " + file_BB, shell=True)

    # read coarse-grained BB bead positions
    with open(file_BB,'r') as fid:
        dat = fid.readlines()
    dat = dat[header_lines:-1]
    print('Number of coarse-grained BB beads in your protein: ' + str(len(dat)))

    indBB = []
    nameAA = []
    for k in range(0, len(dat)):
        tmp = dat[k]
        tmp = [ tmp[0:6],
                tmp[6:11],
                tmp[12:16],
                tmp[16],
                tmp[17:20],
                tmp[21],
                tmp[22:26],
                tmp[26],
                tmp[30:38],
                tmp[38:46],
                tmp[46:54],
```

```
                tmp[54:60],
                tmp[60:66],
                tmp[76:78],
                tmp[78:80],
                ]
        indBB.append([ int(tmp[1]), float(tmp[8]), float(tmp[9]), float(tmp[10]) ])
        nameAA.append(tmp[4])
    indBB = np.array(indBB)

    # read OV contact map
    with open(file_OV,'r') as fid:
        dat = fid.readlines()
    dat = dat[header_lines:-1]
    print('Number of contacts read from your OV contact map file: ' + str(len(dat)))

    map_OVrCSU = []
    row = []
    for k in range(0, len(dat)):
        tmp = dat[k]
        tmp = tmp.replace('\t',' ')
        tmp = tmp.split()
        for l in cols:
            row.append(float(tmp[l]))
        map_OVrCSU.append(row)
        row = []

    # read rCSU contact map
    with open(file_rCSU,'r') as fid:
        dat = fid.readlines()
    dat = dat[header_lines:-1]
    print('Number of contacts read from your rCSU contact map file: ' + str(len(dat)))

    for k in range(0, len(dat)):
        tmp = dat[k]
        tmp = tmp.replace('\t',' ')
        tmp = tmp.split()
        for l in cols:
            row.append(float(tmp[l]))
        map_OVrCSU.append(row)
        row = []

    return indBB, nameAA, map_OVrCSU

def get_go(indBB, nameAA, map_OVrCSU, cutoff_short, cutoff_long, go_eps_intra, go_eps_inter, seqDist, missRes, chain_start, chain_end):
    # calculate the distances based on the coordinates of the CG BB bead
    for k in range(0, len(map_OVrCSU)):
        dist_vec = indBB[ int(map_OVrCSU[k][1])-missRes-1 ,1:4] - indBB[ int(map_OVrCSU[k][0])-missRes-1 ,1:4]
        map_OVrCSU[k][2] = np.linalg.norm(dist_vec) /10     # [Ang] to [nm]

    pairs = []
    for k in range(0, len(map_OVrCSU)):
        if (map_OVrCSU[k][2] > cutoff_short) and (map_OVrCSU[k][2] < cutoff_long) and ( abs(map_OVrCSU[k][1]-map_OVrCSU[k][0]) >= seqDist ):
            intra = 0
            for i in range(len(chain_start)):
                if (chain_start[i] <= int(map_OVrCSU[k][0]) <= chain_end[i] and chain_start[i] <= int(map_OVrCSU[k][1]) <= chain_end[i]):
                    intra += 1    #if intramolecular contact then intra += 1
```

```
                break

        if intra > 0:    #intramolecular contacts
            # parameters for LJ potential
            sigma = map_OVrCSU[k][2] / 1.12246204830        # calc sigma for the LJ potential in [nm]
            Vii = 4.0 * pow(sigma,6) * go_eps_intra
            Wii = 4.0 * pow(sigma,12) * go_eps_intra
            pairs.append([indBB[ int(map_OVrCSU[k][0])-missRes-1 ,0], indBB[ int(map_OVrCSU[k][1])-missRes-1 ,0], Vii, Wii,
                                 map_OVrCSU[k][0], map_OVrCSU[k][1], map_OVrCSU[k][2], sigma])
            Vii = []
            Wii = []
        else:    #intermolecular contacts
            # parameters for LJ potential
            sigma = map_OVrCSU[k][2] / 1.12246204830        # calc sigma for the LJ potential in [nm]
            Vii = 4.0 * pow(sigma,6) * go_eps_inter
            Wii = 4.0 * pow(sigma,12) * go_eps_inter
            pairs.append([indBB[ int(map_OVrCSU[k][0])-missRes-1 ,0], indBB[ int(map_OVrCSU[k][1])-missRes-1 ,0], Vii, Wii,
                                 map_OVrCSU[k][0], map_OVrCSU[k][1], map_OVrCSU[k][2], sigma])
            Vii = []
            Wii = []

    elif map_OVrCSU[k][2] > cutoff_long:
        print('This contanct is excluded due to distance > cutoff_long: ' + str(map_OVrCSU[k]))
    elif map_OVrCSU[k][2] < cutoff_short:
        print('This contanct is excluded due to distance < cutoff_short: ' + str(map_OVrCSU[k]))
    elif abs(map_OVrCSU[k][1]-map_OVrCSU[k][0]) < seqDist:
        print('This contanct is excluded because the AA have less than ' + str(seqDist-1) + ' other AA between each other: '+
              str(map_OVrCSU[k]))

sym_pairs = []
# count contacts only once; exclude asymmetric rCSU contacts (cf. doi 10.1063/1.4929599)
for k in range(0, len(pairs)):
    if pairs[k][0] < pairs[k][1]:
        for l in range(k+1, len(pairs)):
            if (pairs[l][0] == pairs[k][1]) and (pairs[l][1] == pairs[k][0]):
                sym_pairs.append(pairs[k])

print ('- - - -')
print ('These results exclude the contacts with distances higher than the cutoff_long (' + str(cutoff_long) + ' nm), shorter'+
       ' than the cutoff_short (' + str(cutoff_short) + ' nm), or where the AA have less than '+
       str(seqDist-1) + ' other AA between each other:')
print ('Sum of symmetric (doubly counted) and asymmetric OV + rCSU contacts: ' + str(len(pairs)))
print ('Only symmetric OV + rCSU contacts (singly counted):' + str(len(sym_pairs)))

return sym_pairs

def write_files(file_pref, sym_pairs, missAt, indBB, missRes, Natoms, nameAA, go_eps_intra, go_eps_inter, c6c12, chain_start, chain_end):
    # write the interaction table for the Go-like bonds
    with open(file_pref + '_go-table_VirtGoSites1.itp','w') as f:
        f.write('; OV + symmetric rCSU contacts \n')
        if (c6c12 == 1):
            for k in range(0, len(sym_pairs)):
                # to write the LJ potential itp:
                s2print = " %s_%s  %s_%s    1  %.10f  %.10f  ;  %s  %s  %.3f \n" % (file_pref, str(int(sym_pairs[k][4])), file_pref,
                          str(int(sym_pairs[k][5])), sym_pairs[k][2], sym_pairs[k][3], str(int(sym_pairs[k][0]) +missAt),
                          str(int(sym_pairs[k][1]) +missAt), sym_pairs[k][6])
```

```
                    # atom index and residue index adapted due to missing residues
            f.write(s2print)
    else:
        for k in range(0, len(sym_pairs)):
            # to write the LJ potential itp:

            intra = 0
            for i in range(len(chain_start)):
                if (chain_start[i] <= int(sym_pairs[k][4]) <= chain_end[i] and chain_start[i] <= int(sym_pairs[k][5]) <= chain_end[i]):
                    intra += 1
                    break

            if intra > 0:
                s2print = " %s_%s  %s_%s    1  %.10f  %.10f  ;  %s  %s  %.3f \n" % (file_pref, str(int(sym_pairs[k][4])), file_pref,
                          str(int(sym_pairs[k][5])), sym_pairs[k][7], go_eps_intra, str(int(sym_pairs[k][0]) +missAt),
                          str(int(sym_pairs[k][1]) +missAt), sym_pairs[k][6])
                          # atom index and residue index adapted due to missing residues
            else:
                s2print = " %s_%s  %s_%s    1  %.10f  %.10f  ;  %s  %s  %.3f \n" % (file_pref, str(int(sym_pairs[k][4])), file_pref,
                          str(int(sym_pairs[k][5])), sym_pairs[k][7], go_eps_inter, str(int(sym_pairs[k][0]) +missAt),
                          str(int(sym_pairs[k][1]) +missAt), sym_pairs[k][6])
                          # atom index and residue index adapted due to missing residues

            f.write(s2print)

subprocess.call("echo '#include \"" + file_pref + "_go-table_VirtGoSites.itp\"' >> go-table_VirtGoSites.itp ", shell=True)

# write supplementary file: BB virtual particle definitions for martini.itp
with open(file_pref + '_BB-part-def_VirtGoSites1.itp','w') as f:
    f.write('; protein BB virtual particles \n')
    for k in range(0, len(indBB)):
        s2print = "%s_%s 0.0 0.000 A 0.0 0.0 \n" % (file_pref, str(k+1 + missRes)) # residue index adapted due to missing residues
        f.write(s2print)
subprocess.call("echo '#include \"" + file_pref + "_BB-part-def_VirtGoSites.itp\"' >> BB-part-def_VirtGoSites.itp ", shell=True)

# write supplementary file: exclusions for protein.itp
with open(file_pref + '_exclusions_VirtGoSites1.itp','w') as f:
    f.write(';[ exclusions ] \n')
    f.write('; OV + symmetric rCSU contacts \n')
    for k in range(0, len(sym_pairs)):
        s2print = " %s  %s  \t ;  %s  %s \n" % (str(int(sym_pairs[k][0]) +missAt), str(int(sym_pairs[k][1]) +missAt),
                                              str(int(sym_pairs[k][4] -missRes)), str(int(sym_pairs[k][5] -missRes)))
                                   # atom index and residue index adapted due to missing residues
        f.write(s2print)

# write supplementary file: Go-like bonds as harmonic bonds for visulization of the protein
with open(file_pref + '_go4view_harm1.itp','w') as f:
    f.write('; Go bonds as harmonic bonds between the virtual particles: \n')
    f.write('; OV + symmetric rCSU contacts \n')
    for k in range(0, len(sym_pairs)):
        # to write the harmonic bonds itp:
        s2print = " %s  %s  1  %s  1250  ; %s_%s  %s_%s \n" % (str(int(sym_pairs[k][4] +Natoms)), str(int(sym_pairs[k][5] +Natoms)),
                  str(round(sym_pairs[k][6],3)), file_pref, str(int(sym_pairs[k][4] -missRes)), file_pref,
                  str(int(sym_pairs[k][5] -missRes)))
                  # the bonds are added between the virtual particles
        f.write(s2print)
```

```
        for k in range(0, len(indBB)):
            if (np.sum(np.array(sym_pairs)[:,4]==k+1) + np.sum(np.array(sym_pairs)[:,5]==k+1)) == 0:
                s2print = " %s  %s  1  1.  1     ; %s_%s  %s_%s --> added for vmd \n" % (str(int(k+1 +Natoms)), str(int(k +Natoms)),
                          file_pref, str(k+1), file_pref, str(k))
                f.write(s2print)

def main():
    args = user_input()
    file_BB, file_OV, file_rCSU, header_lines, seqDist, cols, missAt, c6c12 = get_settings()

    indBB, nameAA, map_OVrCSU = read_data(args.s, args.f, file_BB, file_OV, file_rCSU, header_lines, cols)

    sym_pairs = get_go(indBB, nameAA, map_OVrCSU, args.cutoff_short, args.cutoff_long, args.go_eps_intra, args.go_eps_inter, seqDist,
                       args.missres, args.chain_start, args.chain_end)

    write_files(args.moltype, sym_pairs, missAt, indBB, args.missres, args.Natoms, nameAA, args.go_eps_intra, args.go_eps_inter,
                c6c12, args.chain_start, args.chain_end)
    print('All symmetric OV and rCSU contacts written! Have fun!')

if __name__ == '__main__':
    main()
```

"Snake Oil" implementation

The script, including instructions and an example system (amyloid-β), can be found in the following GitHub repository:

https://github.com/kkorshunova/multichain-martini-go

References

(1) Poma, A. B.; Cieplak, M.; Theodorakis, P. E. Combining the MARTINI and Structure-Based Coarse-Grained Approaches for the Molecular Dynamics Studies of Conformational Transitions in Proteins. *J. Chem. Theory Comput.* **2017**, *13*, 1366–1374.

(2) Wołek, K.; Gómez-Sicilia, À.; Cieplak, M. Determination of contact maps in proteins: A combination of structural and chemical approaches. *J. Chem. Phys.* **2015**, *143*, 243105.

(3) de Jong, D. H.; Singh, G.; Bennett, W. D.; Arnarez, C.; Wassenaar, T. A.; Schafer, L. V.; Periole, X.; Tieleman, D. P.; Marrink, S. J. Improved parameters for the martini coarse-grained protein force field. *J. Chem. Theory Comput.* **2013**, *9*, 687–697.

(4) Kroon, P. C.; Grünewald, F.; Barnoud, J.; van Tilburg, M.; Souza, P. C.; Wassenaar, T. A.; Marrink, S.-J. Martinize2 and Vermouth: Unified Framework for Topology Generation. *eLife* **2023**, *12*, RP90627.

(5) Souza, P. C.; Thallmair, S.; Marrink, S. J.; Mera-Adasme, R. An Allosteric Pathway in Copper, Zinc Superoxide Dismutase Unravels the Molecular Mechanism of the G93A Amyotrophic Lateral Sclerosis-Linked Mutation. *J. Phys. Chem. Lett.* **2019**, *10*, 7740–7744.

(6) Thallmair, S.; Vainikka, P. A.; Marrink, S. J. Lipid Fingerprints and Cofactor Dynamics of Light-Harvesting Complex II in Different Membranes. *Biophys. J.* **2019**, *116*, 1446–1455.

(7) GROMACS 2020.6. 2020; `https://manual.gromacs.org/documentation/2020.6/index.html`.

(8) Abraham, M. J.; Murtola, T.; Schulz, R.; Páll, S.; Smith, J. C.; Hess, B.; Lindahl, E. Gromacs: High performance molecular simulations through multi-level parallelism from laptops to supercomputers. *SoftwareX* **2015**, *1*, 19–25.

(9) Van Der Spoel, D.; Lindahl, E.; Hess, B.; Groenhof, G.; Mark, A. E.; Berendsen, H. J. GROMACS: Fast, flexible, and free. *J. Comput. Chem.* **2005**, *26*, 1701–1718.

(10) Abraham, M. et al. GROMACS 2023 Manual. 2023; https://zenodo.org/records/7588711.
